# Mechanisms of intron gain and loss in Drosophila

**DOI:** 10.1186/1471-2148-11-364

**Published:** 2011-12-19

**Authors:** Paul Yenerall, Bradlee Krupa, Leming Zhou

**Affiliations:** 1 Department of Biological Sciences, University of Pittsburgh, Pittsburgh, PA 15260, USA; 2 Department of Computer Science, University of Pittsburgh, Pittsburgh, PA 15260, USA; 3 Department of Health Information Management, University of Pittsburgh, Pittsburgh, PA 15260, USA; 4 Department of Bioengineering, University of Pittsburgh, Pittsburgh, PA 15260, USA

## Abstract

**Background:**

It is widely accepted that orthologous genes have lost or gained introns throughout evolution. However, the specific mechanisms that generate these changes have proved elusive. Introns are known to affect nearly every level of gene expression. Therefore, understanding their mechanism of evolution after their initial fixation in eukaryotes is pertinent to understanding the means by which organisms develop greater regulation and complexity.

**Results:**

To investigate possible mechanisms of intron gain and loss, we identified 189 intron gain and 297 intron loss events among 11 Drosophila species. We then investigated these events for signatures of previously proposed mechanisms of intron gain and loss. This work constitutes the first comprehensive study into the specific mechanisms that may generate intron gains and losses in Drosophila. We report evidence of intron gain via transposon insertion; the first intron loss that may have occurred via non-homologous end joining; intron gains via the repair of a double strand break; evidence of intron sliding; and evidence that internal or 5' introns may not frequently be deleted via the self-priming of reverse transcription during mRNA-mediated intron loss. Our data also suggest that the transcription process may promote or result in intron gain.

**Conclusion:**

Our findings support the occurrence of intron gain via transposon insertion, repair of double strand breaks, as well as intron loss via non-homologous end joining. Furthermore, our data suggest that intron gain may be enabled by or due to transcription, and we shed further light on the exact mechanism of mRNA-mediated intron loss.

## Background

Spliceosomal introns, segments of RNA that are excised by the spliceosome during the processing of pre-mRNA in eukaryotes, are found in varying quantities and positions among orthologous genes. By identifying orthologs, aligning gene sequences, and coupling intron absences/presences with known species phylogenies, numerous studies have identified the number of intron gains and losses that have occurred among species throughout evolution [1-11]. However, very little is known about the molecular mechanisms underlying these changes [12,13].

As a deeper understanding of gene expression emerges, it is evident that introns not only increase proteome diversity through their well known role in alternative splicing [14], but also influence every stage of pre-translational gene expression [15]. Important regulatory elements such as miRNAs and snoRNAs are commonly found within introns in animals [16], and recently introns in the human genome have been shown to harbor thousands of non-coding RNAs, key regulators of gene expression [17]. The splicing process alone has been shown to increase transcriptional efficiency and the nuclear export of transcripts [15,18-21]. Therefore, understanding the molecular mechanisms that create and remove introns provides insight into one of the mechanisms by which eukaryotic organisms develop greater regulation and complexity.

Two previously hypothesized mechanisms of intron loss are *Reverse Transcriptase-Mediated Intron Loss *(referred to as *RTMIL *in this work) [22] and *Genomic Deletions*. RTMIL occurs when cDNA, either directly or after retroposition into the genome, recombines with an intron-present gene, resulting in the precise deletion of intron(s) [23]. Genomic deletions are general genomic deletion events that, by chance, delete an intron [24]. Therefore, the genomic deletion of introns may occur via various molecular mechanisms and may produce precise or imprecise intron losses. Recently, double strand break repair (DSBR) by non-homologous end joining (NHEJ) has been implicated as a common means for the genomic deletion of introns [25]. RTMIL has been demonstrated in yeast [26,27], and general genomic deletions are known to occur. However, the prevalence of each proposed mechanism of intron loss is unknown.

Previously hypothesized mechanisms of intron gain include: *Intron Transposition *[28], in which an intron transposes or "reverse splices" into a previously intronless position in a transcript, and this transcript is then reverse transcribed and recombined with the original gene; *Transposon Insertion *[29], in which a transposon inserts into a gene and forms a spliceable intron; *Tandem Genomic Duplications *[30], in which the tandem duplication of a gene segment creates a spliceable intron; *Intron Transfer *[31], in which a paralog transfers an intron via gene conversion to an intron-absent position; *Insertion of a Group II Intron *[28], in which a group II intron (a type of intron known to reverse splice or retrohome in some organelle genomes) inserts into a nuclear gene and creates a spliceosomal intron; *Intron Gain During Double Strand Break Repair *[4], in which a DNA segment that may function as a spliceable intron is inserted during DSBR; and *Intronization *[32,33], in which mutations in exonic sequence produce functional splice signals, forming a new intron with previously exonic sequence.

Unlike most mechanisms of intron gain and loss which involve the insertion or deletion of DNA segments, *Intron Sliding *[34-36] has been hypothesized to present the appearance of concurrent intron loss and gain without removing or inserting DNA. This may occur when orthologous introns "slide" through a gene, while leaving the coding sequence largely unaffected. If the intron slides far enough from its original position, it may appear as if a gene has both lost and gained an intron. Evidence of intron sliding in Drosophila exists [34]; however, there is debate over the viability of this mechanism [35-38].

Out of all the proposed mechanisms of intron gain and loss, only RTMIL has been shown to occur *in vivo *[26,27]. Therefore, in order to find support for the occurrence of other proposed mechanisms of intron gain or loss, researchers have attempted to identify intron gains or losses that appear to have occurred via a specific mechanism. Evidence has been found to support the occurrence of: intron loss due to genomic deletions in Drosophila and Pufferfish [39,40]; intron gain by intron transposition in *Oikopleura *[5]; intron gain by transposon insertion in maize, rice and *Oikopleura *[5,41,42]; intron gain by intron transfer in *Chironomus thummi *and *Aspergillus *fungi [11,31]; intron gain by tandem genomic duplications in a multitude of eukaryotes [34,43,44]; intron gain during DSBR in *Daphnia pulex *and *Aspergillus *fungi [4,11]; intron gain by intronization in *Cryptococcus *and *Caenorhabditis *[33,37]; and intron sliding in Drosophila [34]. However, these findings are insufficient to prove the existence of any proposed mechanism. In order to determine if these proposed mechanisms of intron gain or loss are universal mechanisms operating in all eukaryotes, as opposed to either singular events or mechanisms that only occur in a few species, multiple unambiguous instances of each mechanism must be located in all eukaryotic kingdoms.

Only a few of the proposed mechanisms of intron gain or loss have been shown to occur in Drosophila [6,34,39]. Therefore, we chose to investigate the ability of all proposed mechanisms to operate in Drosophila. To this end, we first identified high confidence cases of intron gains and losses among 11 Drosophila species (*D. melanogaster*, *D. pseudoobscura*, *D*. *virilis*, *D. sechellia*, *D. yakuba*, *D. erecta*, *D. ananassae*, *D. persimilis*, *D. willistoni*, *D. mojavensis*, and *D. grimshawi*). We then analyzed these events extensively for signatures of previously proposed mechanisms of intron gain and loss. These 11 well-sequenced and well-annotated Drosophila species enabled us to identify intron gains and losses that have occurred relatively recently (2-40 million years ago) [45]. This fine time scale allowed us to analyze these events before extensive sequence divergence may have occurred, which has the potential to disguise the mechanism(s) underlying these events.

## Results

**Figure 1:**
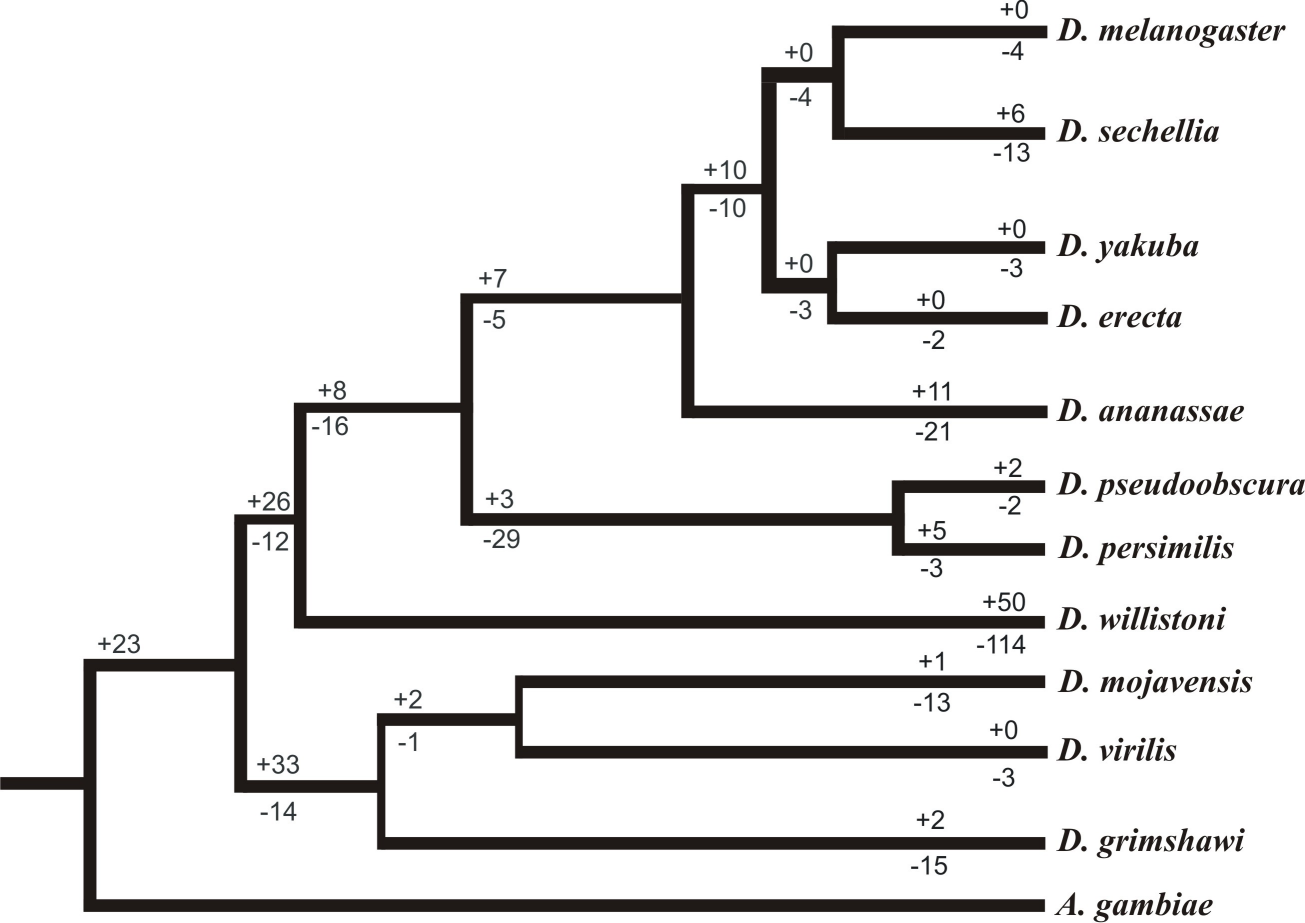
**Drosophila phylogenetic tree illustrating the numbers of intron gains and losses**. Pluses indicate the number of gained introns; minuses indicate the number of lost introns. Numbers at far right of the tree represent events identified in one species. Numbers at nodes represent events assumed to have occurred in ancestors. Branch lengths are drawn roughly to scale and do not indicate precise evolutionary distances. A larger phylogenetic tree drawn to scale (with the number of intron gains and losses mapped onto the tree) can be found in Additional file 1, Figure S1.

**Figure 2:**
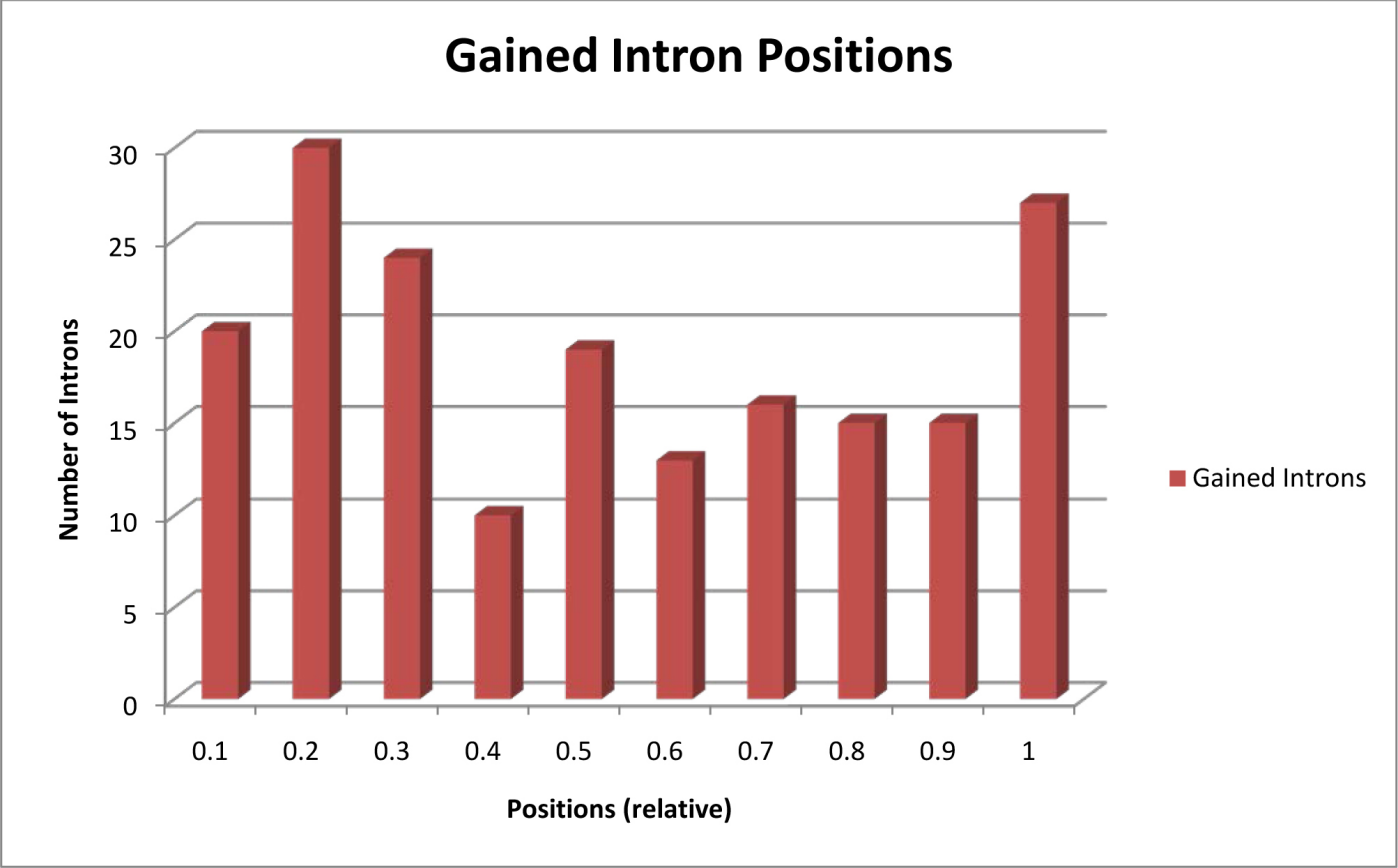
**Histogram of positions of gained introns**. Histogram displaying the relative position (i.e. on a scale of 1) of intron gains in the gene.

Within the final dataset of 353 orthologs, we identified 189 intron gains and 287 intron losses with 112 gains and 94 losses located at ancestral nodes (Figure 1) and 77 gains and 193 losses located within a single species (Table 1). Using a different dataset, we support previous findings of widespread heterogeneity in the rates of intron gain and loss among Drosophila species [6]. Overall, in comparison to introns from all 11 Drosophila species (59% AT content, average size 1015 bp), gained introns were of similar composition but shorter in length (64% AT content, average size 398 bp). Additionally, in accordance with previous research in Drosophila [1,6], these gained introns were biased towards the 5' end of genes (Kolmogorov-Smirnov test, p = 0.0421, Figure 2).

**Table 1:** Information about each species and the number of intron gains and losses found within each species

Species Name	Assembled Genome Size	Protein Coding Genes	Number of Introns	Average Intron Size(bp)	Introns Analyzed	Gained Introns	Lost Introns
*D. melanogaster*	118 Mb	13, 919	53, 459	1, 482	1, 401	0	4

*D. sechellia*	115 Mb	16, 467	41, 655	799	1, 391	6	13

*D. yakuba*	127 Mb	16, 077	42, 642	824	1, 392	0	3

*D. erecta*	134 Mb	15, 044	40, 986	835	1, 397	0	2

*D. ananassae*	176 Mb	15, 069	41, 345	1, 026	1, 391	11	21

*D. pseudoobscura*	127 Mb	16, 062	41, 804	823	1, 372	2	2

*D. persimilis*	138 Mb	16, 874	41, 743	949	1, 370	5	3

*D. willistoni*	187 Mb	15, 512	40, 896	1, 203	1, 338	50	114

*D. mojavensis*	161 Mb	14, 594	40, 199	1, 075	1, 391	1	13

*D. virilis*	172 Mb	14, 491	40, 386	1, 071	1, 421	0	3

*D. grimshawi*	138 Mb	15, 585	41, 370	965	1, 396	2	15

### Mechanisms of Intron Loss

#### Reverse Transcriptase-Mediated Intron Loss

**Figure 3:**
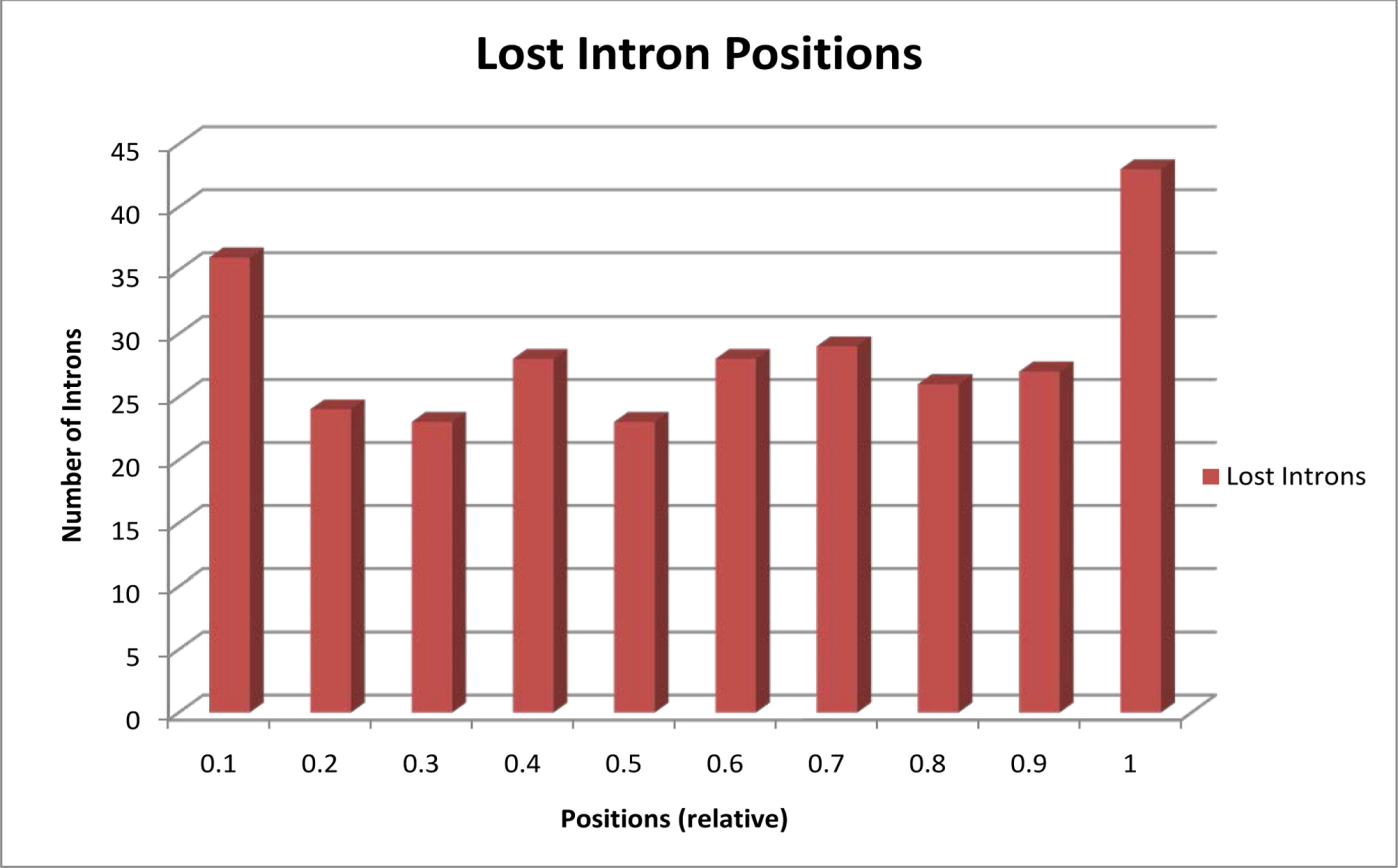
**Histogram of positions of lost introns**. Histogram displaying the relative position (i.e. on a scale of 1) of intron losses in the gene.

Because RTMIL leaves behind no distinct mechanistic signatures, it is only possible to determine its prevalence by analyzing intron deletion biases. These biases arise due to the involvement of reverse transcriptase during RTMIL. Reverse transcriptase has been proposed to be primed on the poly(A) tail of mRNA [22] and transcribe from the 3' end to the 5' end of mRNA. However, reverse transcriptase may not always reach the 5' end of mRNA [46]. Therefore, if intron deletions have commonly occurred via RTMIL, intron deletions are expected to be biased towards the 3' end of genes. Some researchers have identified this bias [1,2,24,44], but others have not [5,6,9,40,42]. Previous reports on the distribution of intron loss positions in Drosophila have been conflicting [1,6]. We found lost intron positions to be uniformly distributed throughout the length of genes that experienced intron loss(es) (Kolmogorov-Smirnov test, p = 0.2112, Figure 3). Other hypothesized mechanistic pathways of RTMIL, whereby RTMIL may delete internal or 5' introns without deleting 3' introns [44,47], may explain this distribution. Alternatively, RTMIL may have not deleted the majority of lost introns in our dataset.

Because RTMIL is transcript-mediated, if RTMIL was a frequent mechanism of intron loss, genes that have lost introns should commonly be germline expressed [23]. To test this assumption, we extracted the *D. mel *ortholog of each gene that experienced an intron loss from our dataset. We then checked these orthologs for moderate germline expression using data downloaded from Flybase [45], the modENCODE project [48], and FlyAtlas [49]. Using this dataset, 187 out of the 287 genes that experienced intron loss were shown to have moderate germline expression. In comparison to the frequency in which we found genes to be germline expressed in *D. melanogaster *(7, 212 out of 13, 752), we found a significant bias for genes that experienced intron loss to be germline expressed (Pearson chi-square test, p < 0.05).

Another deletion bias expected if RTMIL has commonly deleted introns is the frequent loss of adjacent introns. Previous investigations have found adjacent introns to be lost more commonly than would be expected purely by chance [1,11,24,50]. Our dataset contained a total of 9 adjacent intron losses that appear to have occurred simultaneously in the genes *Dwil\GK21739*, *Dsec\GM16466*, and *Dwil\GK24430*. We would have expected 2.7 adjacent intron losses to have occurred purely by chance [1]. Therefore, our dataset show a significant bias for adjacent introns to be lost (Pearson chi-square test, p < 0.05).

In one gene that experienced adjacent intron losses, *Dwil\GK24430*, the first and last introns were conserved while two internal introns were lost. Because these losses were adjacent and appear to have occurred simultaneously, we assume these introns were deleted by RTMIL. The exact mechanism by which RTMIL may remove internal or 5' intron(s) but conserve 3' intron(s) has proved elusive but received considerable attention [9,11,13,44,51,52]. The most commonly proposed mechanism to account for internal or 5' intron loss(es) by RTMIL is the formation of a double stranded mRNA secondary structure upstream from the 3' conserved intron position(s). This secondary structure then "self-primes" reverse transcription during RTMIL, excluding the conserved intron position(s) from reverse transcription and subsequent recombination (i.e. intron loss) [9,44,51,52]. Because the ortholog of *Dwil\GK24430 *in *D*. *melanogaster*, *elgi*, was shown to have high expression levels in the ovaries of adult flies [48] and orthologs of *Dwil\GK24430 *have highly similar sequences (which suggests that the coding sequence has been conserved), *Dwil\GK24430 *was investigated for the ability to have self-primed reverse transcription during RTMIL. We determined the 5' and 3' untranslated regions (UTRs) of *Dwil\GK24430 *using the Augustus program [53], determined the polyadenylation site using PolyAPred [54], appended poly(A) tails of various lengths, and ran these predicted mRNA sequences through the RNA folding program mfold [55]. All predicted secondary structures could not account for the pattern of intron losses that occurred in *Dwil\GK24430*. Therefore, it is not likely that the self-priming of reverse transcription during RTMIL accounted for these internal intron losses.

#### Genomic Deletions

**Figure 4:**
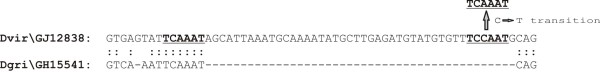
**Genomic deletion of an intron by NHEJ**. Alignment of intron 1 in *Dvir\GJ12838 *with unaligned nucleotides from the coding sequence of *Dgri\GH15541*, which experienced an intron loss at this position. Direct repeats (bolded and underlined) may have been used for microhomology directed ligation during NHEJ. The second cyostine in the downstream repeat may have undergone a C→T transition.

Similar to intron loss via RTMIL, the precise genomic deletion of an intron is difficult to confidently detect after its occurrence. Therefore, we identified imprecise intron losses. To locate imprecise losses, we examined the former intron-exon junctions of all lost introns. If the intron deletion event appeared to have inserted nucleotides into the coding sequence of the gene, these inserted nucleotides were extracted and compared to conserved orthologous introns using the FASTA program [56]. Using this method we identified an imprecise intron loss that may have occurred via NHEJ (Figure 4), a recently hypothesized mechanism of intron loss [25]. Direct repeats that likely flanked this intron prior to deletion may have mediated deletion by providing a recessed microhomology for efficient ligation during NHEJ [57].

Because introns flanked by direct repeats have been hypothesized to be preferentially deleted via genomic deletions [47], it is expected that throughout evolution, introns flanked by direct repeats will be preferentially lost. Therefore, in an attempt to determine the prevalence of intron loss via genomic deletions in our dataset, for each intron loss identified within a single species we searched the intron-exon junctions of the closest (in evolutionary distance) conserved orthologous intron for the presence of direct repeats ≥ 5 bp in length. In our dataset, 27% of these introns were flanked by direct repeats, nearly identical to the percent of direct repeats found flanking 100 randomly selected conserved introns (26%). This suggests that RTMIL may have deleted the majority of introns in our dataset. However, it is possible that sequence divergence throughout evolution may have eliminated many direct repeats that originally flanked these conserved orthologous introns.

### Mechanisms of Intron Gain

#### Transposon Insertion

**Figure 5:**
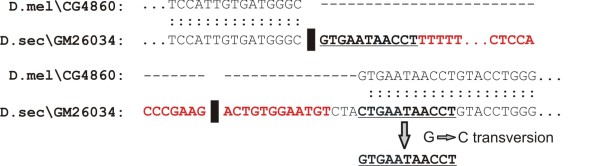
**Intron gain via transposon insertion**. The solid black bar indicates intron-exon junctions. Red nucleotides indicate matched nucleotides between *Doc1053 *and intron 3 in *Dsec\GM26034*. Bolded and underlined nucleotides represent TSDs caused by insertion of the transposon. The first nucleotide in the downstream TSD likely underwent a G→C transversion. The insertion of *Doc1053 *did not change the reading frame of *Dsec\GM26034 *but did insert five amino acids (Thr, Met, Ser, Thr, and Glu).

To identify intron gains that occurred via transposon insertion, all gained intronic sequences were compared to the canonical transposon sequences from Flybase [45] using the FASTA program [56]. A hit between the third intron in *Dsec\GM26034 *and the retrotransposon *Doc1053 *occurred with 98.4% similarity and 99% coverage. Target site duplications (TSDs) are located at the 5' end and 15 nucleotides downstream from the end of this intron, indicating that the insertion of *Doc1053 *alone resulted in intron gain (Figure 5).

#### Double Strand Break Repair

**Figure 6:**
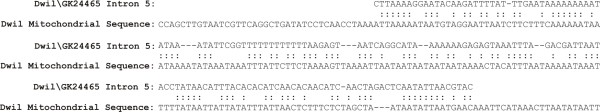
**Intron gain by DSBR**. Alignment between the reverse complement of a gained intron in *Dwil\GK24465 *and a segment of *D. wil*'s mitochondria (e-value = 0.032, coverage = 99%). The best score produced by randomly shuffling and realigning these sequences 1, 000 times was significantly lower (Pearson chi-square test, p < 0.05) than the score between the original sequences.

DSBR has recently been proposed to result in intron gain if NHEJ inserts filler DNA that may function as a spliceable intron [4,58]. It has been shown that this filler DNA may be preferentially of mitochondrial origin [59,60]. To identify cases of intron gain that occurred via the repair of double strand breaks, we compared all gained intronic sequences to their respective nuclear and mitochondrial genomes. Eight gained introns (*Dwil\GK13533 *intron 2, *Dana\GF12884 *intron 3, *Dper\GL20060 *intron 2, *Dmel\CG9297 *intron 4, *Dmoj\GI21017 *intron 5, *Dmoj\GI21017 *intron 6, *Dvir\GJ24248 *intron 8, and *Dwil\GK24841 *intron 1, average length = 113 bp) matched to their respective mitochondrial genomes with ≥ 90% query sequence coverage and e-value ≤ 0.1, suggesting that these introns may have been inserted via NHEJ. One of these introns, *Dwil\GK24465 *intron 5, displayed significant similarity to a mitochondrial sequence (Figure 6). Many other hits were found with lower coverage levels (60-70%) but better e-values (e ≤ 10^-4^).

Because direct repeats frequently flank filler DNA inserted via NHEJ [61], to determine the prevalence of intron gain via NHEJ in our dataset we searched the intron-exon junctions of all gained introns for direct repeats of length ≥ 5 bp. We identified direct repeats flanking 19 out of 77 gained introns; however, in comparison to a random set of 100 conserved introns (26 of which were flanked by direct repeats), this level did not reach statistical significance. This suggests two possibilities. One is that direct repeats may not commonly flank DNA inserted by NHEJ in Drosophila, as the frequency and size of direct repeats inserted by NHEJ when using filler DNA has been shown to vary in different organisms and cell types [61-63]. Alternatively, NHEJ may not be a common mechanism of intron gain in Drosophila.

#### Transcription-Mediated Intron Gain?

We did not identify any intron gains that occurred via intron transposition in our dataset, the only proposed transcript-mediated mechanism of intron gain. However, genes that have experienced intron gains are highly overrepresented in our germline expression dataset (135 out of 189, Pearson chi-square test, p < 0.01), similar to findings in *Caenorhabditis *[10]. This overrepresentation of germline expression in genes that have experienced intron gain suggests that intron gain may be enabled by or due to transcription.

### INTRON SLIDING

Intron sliding, the sliding or relocation of orthologous introns, has been proposed to be a rare event that may move introns very small distances [35,37,38]. We identified 4 introns that appear to have slid more than 10 bp while leaving the coding sequence largely unaffected. To ensure that these were *bona fide *cases of intron sliding, as opposed to concurrent intron losses and gains, we compared the sequence of introns that appeared to have slid to the sequence of their closest (in evolutionary distance) suspected orthologous introns. Three cases of intron sliding displayed moderate similarity between these introns (e-value ≤ 0.1), while one, the fourth intron in *Dwil\GK22863*, displayed significant similarity to its suspected ortholog intron, intron four in *Dper\GL17458 *(Additional file 1, Figure S2), indicating that this intron experienced intron sliding.

## Discussion

Prior investigations into intron gain and loss in Drosophila [1,6] have yielded different results from the ones presented here. Our results differ greatly from those of Coulombe-Huntington and Majewski [1], who reported intron loss to be much more prominent than intron gain in Drosophila. This difference can be attributed to different methodology and datasets. Coulombe-Huntington and Majewski mapped splice site junctions from *D. melanogaster *onto the other 10 Drosophila species used in this study, whereas we used high quality, full genome annotations produced by the Drosophila research community [45] for the 11 species. As Coulombe-Huntington and Majewski noted, their methodology did not detect events that had occurred in the other 10 Drosophila species, and was therefore unable to detect intron gain events that had occurred in other species. Our results are also slightly different from those of Farlow et al. [6]. This is likely due to different methods of gene annotation in Drosophila species other than *D. melanogaster*. Farlow et al.'s annotations primarily relied upon GeneWise [64], whereas the annotations employed here were produced using a compilation of various *ab initio *and extrinsic methods [45]. This produced markedly different ortholog datasets; only 734 of our initial 1, 611 orthologs overlap between these two studies. Other differences include our use of a distant outlier, *A. gambiae*, which greatly increased the power of Dollo parsimony at peripheral branches, and our inclusion of *D. sechellia *and *D. persimilis*. Finally, it should be noted that the stringent criteria employed here was designed specifically to eliminate the maximal amount of false-positive intron gain and loss events, rather than to identify the precise number of intron gain and loss events among the Drosophila species. Therefore, the number of intron gains and losses reported here may not necessarily reflect the rate of intron turnover in Drosophila.

Our analyses suggest that intron loss frequently occurs via RTMIL in Drosophila. Adjacent introns were lost more frequently than would be expected purely by chance, and genes experiencing intron loss were commonly germline expressed. However, intron deletions were not biased towards the 3' end of genes (Figure 3), as would be expected if RTMIL deleted the majority of introns. Nonetheless, we did not find evidence suggesting that introns were frequently lost via the precise genomic deletion of introns. There are a number of proposed mechanisms that may explain 5' or internal intron loss by RTMIL without the loss of 3' intron(s). Our data suggest that the most commonly proposed mechanism, the self-priming of reverse transcription during RTMIL [9,44,51,52], may not frequently produce internal intron losses via RTMIL in Drosophila. An alternative explanation for 5' or internal intron loss by RTMIL without the loss of 3' intron(s) was proposed by Sharpton et al. in *C. elegans*. Researchers elegantly demonstrated that genes experiencing two or more 3' intron losses (presumably by RTMIL) are preferentially recombined during meiosis at their 3' ends with alleles that have not experienced intron loss [44]. This may have accounted for the uniform distribution of intron losses found in this study in Drosophila (Figure 3).

A recent study suggested that NHEJ may play a prominent role in both intron gain and loss [25], and our investigation in Drosophila supports this idea. Similar to previous research [4,11], we identified intron gains that likely occurred via NHEJ using mitochondrial DNA (an example is shown in Figure 6). We also identified the first case of an intron loss that may have occurred via NHEJ (Figure 4). The ability of NHEJ to both create and remove introns suggests an interesting scenario in intron evolution: introns gained by NHEJ may commonly be flanked by direct repeats [61], and introns flanked by direct repeats may be preferentially deleted by NHEJ [47,57]. This may be a mechanism by which new introns are "screened" for selective advantages. Under selection pressure, new introns that provide an advantage to the species may be conserved, whereas those that do not may be lost.

For mechanisms of intron gain, we identified an intron gain that unambiguously occurred via the insertion of a transposable element (Figure 5). In combination with previous findings of intron gain via transposon insertion in maize, rice, and *Oikopleura *[5,41,42], this strongly suggests that transposons may create novel introns in all eukaryotes that harbor active transposons.

In our dataset, 187 gained introns do not appear to have been definitively created by any of the proposed mechanisms of intron gain. It is possible that sequence divergence has obscured the source of some of these introns. However, this finding is perplexing, especially for the 7 gained introns found between *D. per *and *D. pse*, which likely radiated only 2 million years ago [65]. We identified a significant bias for genes that have experienced intron gain to be germline expressed, which suggests that transcription may play a prominent role in intron gain. Nonetheless, we find no evidence of intron gain via intron transposition, the only proposed transcript-mediated mechanism of intron gain. Furthermore, intron gains in Drosophila are biased towards the 5' end of genes (Figure 2) [1,6], indicating that reverse transcription may not play a significant role in intron gain. This is further supported by a recent investigation into the role of reverse transcriptase in intron gain and loss [66]. Together, these findings suggest that the act of transcription itself may promote or cause intron gain. We speculate that this may be due to transcription-associated recombination (TAR). TAR generally uses homologous recombination [67]; however, TAR has been shown to occasionally use non-homologous recombination [68,69] and is functionally different from homology-directed DSBR [70]. It is therefore possible that TAR may occasionally insert DNA segments that function as introns. However, a deeper understanding of TAR, which is still poorly characterized, is necessary to fully explore this possibility. Alternatively, uncharacterized errors by or interactions with the transcriptional machinery may facilitate or result in intron gain.

Finally, we identified one unambiguous case of intron sliding in Drosophila. A previous investigation that located near intron pairs also found evidence of intron sliding in Drosophila [34]. This report, in combination with our findings, strongly suggests that intron sliding occurs in Drosophila. However, we do note that intron sliding does not appear to occur in all organisms [35,37]. Therefore, further research into the possibility of this mechanism to operate in other species is necessary.

## Conclusion

The use of 11 well-annotated Drosophila species and an annotated outlier, *A. gam*, as well as the strict criteria used to identify intron gains and losses, likely produced a low false-positive rate. Publicly available data for Drosophila - such as mitochondrial genome sequences, extensive expression data, and a well-characterized transposon set - provided us with excellent tools to determine if intron gains or losses occurred via any previously proposed mechanisms. Combined, this data enabled us to identify intron gains that occurred via transposon insertion and double strand break repair. Furthermore, our data suggest that transcription may promote or occasionally cause intron gain. We speculate that this may occur via TAR or uncharacterized errors by or interactions with the transcriptional machinery. However, the definitive mechanism by which this may occur eludes us and awaits further investigation.

As research progresses, the exact molecular mechanisms of intron loss are becoming more clear. Our data suggest that RTMIL was responsible for the majority of intron losses identified in this study. However, we also found evidence suggesting that the self-priming of reverse transcription during RTMIL may not occur. It is likely that a different hypothesis may account for internal or 5' intron losses via RTMIL [44]. We also identified the first case of intron loss that may have occurred via NHEJ (Figure 4) and speculate that the ability of NHEJ to both generate and delete introns may act as a "screening" mechanism for new introns. Finally, we identified one unambiguous case of the controversial mechanism of intron sliding.

In order to identify and fully understand the molecular mechanisms of intron gain and loss, further research into the ability of proposed mechanisms to operate in other species is necessary. It is likely that different mechanisms operate with varying intensities in different species. Consequently, the use of various species increases the chances of detecting these events. Also, demonstration of these mechanisms in multiple eukaryotic kingdoms is necessary to determine whether these are common mechanisms of intron gain or loss, singular events, or mechanisms that occur in only one species. Investigations at the population level may prove particularly fruitful as they will likely identify events before sequence divergence may obscure their mechanistic origin. Furthermore, it would be even better if *in vitro *or *in vivo *experiments can be designed and conducted to verify these mechanisms. For example, a recent *in vivo *study found that the insertion of a group II intron into a nuclear gene abolishes gene expression [71], strongly suggesting that group II introns no longer create spliceosomal introns. An interesting assay for future research would be to test the ability of NHEJ to delete or insert introns by continuously inducing a double strand break under certain conditions.

## Methods

### Obtaining Orthologs

Most data files (transposons, chromosomes, gene regions, coding regions, intron sequences and annotation files) for the 11 Drosophila species investigated were downloaded from Flybase (release FB2011_01) [45]. Mitochondrial genomes were obtained from GenBank [GenBank: NC_005780, NC_001322, NC_001709, BK006335-BK006341]. To ascertain orthologous genes, an all-against-all comparison among coding sequences of all 11 species was performed using the FASTA program [56]. Only reciprocal best hits with e-value ≤ 10^-30^, similarity ≥ 70% and query sequence coverage ≥ 80% were selected and used to construct an orthologous gene matrix. Considerable debate exists as to the best method of ortholog detection; however, we chose to identify orthologs using reciprocal best hits as this has been shown to produce very low false-positive rates [72]. This process yielded 1, 611 orthologs. Orthologs lacking introns in all 11 species were discarded, yielding a matrix of 1, 405 orthologs. The orthologs in this matrix are 97% identical to Flybase's ortholog dataset. The 9 genes that did not match to Flybase's ortholog dataset did not experience any intron gain or loss events and therefore did not affect our final results.

### Generating Alignments

Artificial introns composed of 30 X's were insertd into intronic positions in each coding sequence and each group of orthologs was globally aligned using the ClustalW program [73] with gap open penalty 80, gap extension penalty 0, gap separation penalty 10 and transition weight 1. An example of a global alignment using artificial introns is shown in Additional file 1, Figure S3. Homogenous artificial introns of length 30 were used for two reasons: they assign consistent weight to each intron position during alignment and produce alignments that are easily readable for further analyses. An *ad hoc *program was then created to locate orthologous introns and convert each alignment into an intron absence/presence (0/1) matrix. All alignments were manually inspected for sequence identity flanking intron positions. If the alignment flanking an intron had a low similarity level, the corresponding 0/1 column in the matrix was deleted, removing these intron(s) from further analyses (an example of an excluded intron is shown in Additional file 1, Figure S4). This criterion eliminated 1006 multiple sequence alignments, leaving 399 alignments for further analyses.

### Identifying Intron Gains and Losses

All multiple sequence alignments were then categorized into 2 groups: those that had discordant intron presences/absences nested within the 11 Drosophila species (Group A, 252 alignments) and those that did not (Group B, 147 alignments). For Group B, if possible an ortholog in *Anopheles gambiae *(*A. gam*) was located to be used as an outlier. *A. gam*'s genome was downloaded from the UCSC genome browser [74] and mRNA sequences were downloaded from the RefSeq database [GenBank:PRJNA163] [75]. The annotation of *A. gam *was generated by mapping mRNA sequences back onto *A. gam*' was generated by mapping mRNA sequences back onto *A*. *gams*'s genome using the program ESTMapper [76]. Orthologs were identified using the FASTA program and extracting reciprocal best hits with e-value ≤ 10^-30^, similarity ≥ 60% and query sequence coverage ≥ 60%. If an ortholog was found in *A. gam*, alignments in Group B were regenerated and reexamined. For alignments in Group B, if no ortholog could be located in *A. gam*, the alignment was excluded. This criterion removed 46 alignments, resulting in the final dataset of 353 multiple sequence alignments (see Additional file 2 for all orthologs used in final analyses). Intron absence/presence matrices for both Group A and B were then processed separately through the program Malin [77] to identify intron gains and losses using Dollo parsimony. Example alignments of intron gains, losses, and alignments that required the outlier *A. gam *can be found in Additional file 1, Figures S5-S9.

### Intron Quality Controls

The ability to accurately identify intron gains and losses relies upon accurate gene annotation. The multitude of comparative and *ab initio *gene finding programs that were used to annotate genes in the 11 Drosophila genomes and the use of well annotated *D. melanogaster *genes during the annotation of the other 10 Drosophila genomes greatly increased the reliability of these annotations [78]. However, since some annotations in the Drosophila species other than *D. melanogaster *may lack experimental validation, annotation errors may exist. Therefore, we applied quality controls to each intron identified as an intron gain in a single species. First, we excluded all intron gains located within a single species that were length 3 n (where "n" is an integer) and did not contain a premature termination codon (PTC) (i.e. DNA segments that, if included in the predicted transcript, would not be expected to elicit nonsense-mediated decay). This criterion was based on a recent study in Drosophila that also used computationally annotated introns to identify intron gains and losses. In that study, 86% of predicted intron gains that were located in a single species and were length 3 n without PTCs were annotation errors as opposed to novel introns [6]. Secondly, we removed all intron gains located in a single species with noncanonical splice sites. Ancestral intron gains (intron gains found in more than one species) and intron losses were not subject to increased scrutiny as the detection of these events is relatively straightforward.

## Authors' contributions

PY participated in the design of the study, performed and conceived analyses, and drafted the manuscript. BK wrote computer programs for data analysis. LZ conceived of the study, participated in its design, wrote computer programs for data analysis and helped to draft the manuscript. All authors read and approved of this version of the manuscript.

## Supplementary Material

Additional file 1 **Supplementary figures**. Figures used to provide further information about the alignments and various cases of intron gain/loss events. Click here for file

Additional file 2 **Ortholog dataset**. A matrix of all orthologs used in final analyses. Each ortholog group is listed on one line. Click here for file
