# Supplementary material for: Mechanisms of intron gain and loss in Drosophila
Source: BMC Evol Biol. 2011 Dec 19;11:364. doi: 10.1186/1471-2148-11-364 (PMC3296678; doi:10.1186/1471-2148-11-364)
Supplement: Additional file 1 — Supplementary figures. Figures used to provide further information about the alignments and various cases of intron gain/loss events. [file 1471-2148-11-364-S1.PDF]

Figure S1. Drosophila phylogenetic tree drawn to scale illustrating the number of intron gains and losses. Pluses indicate the number of gained introns; minuses indicate the number of lost introns. Numbers at far right of the tree represent events identified in one species. Numbers at nodes represent events assumed to have occurred in ancestors. All branch lengths are drawn to scale except for the branch leading to *A. gam*, which likely radiated from the common ancestor of Drosophila and *A. gam* 250 million years ago [1].

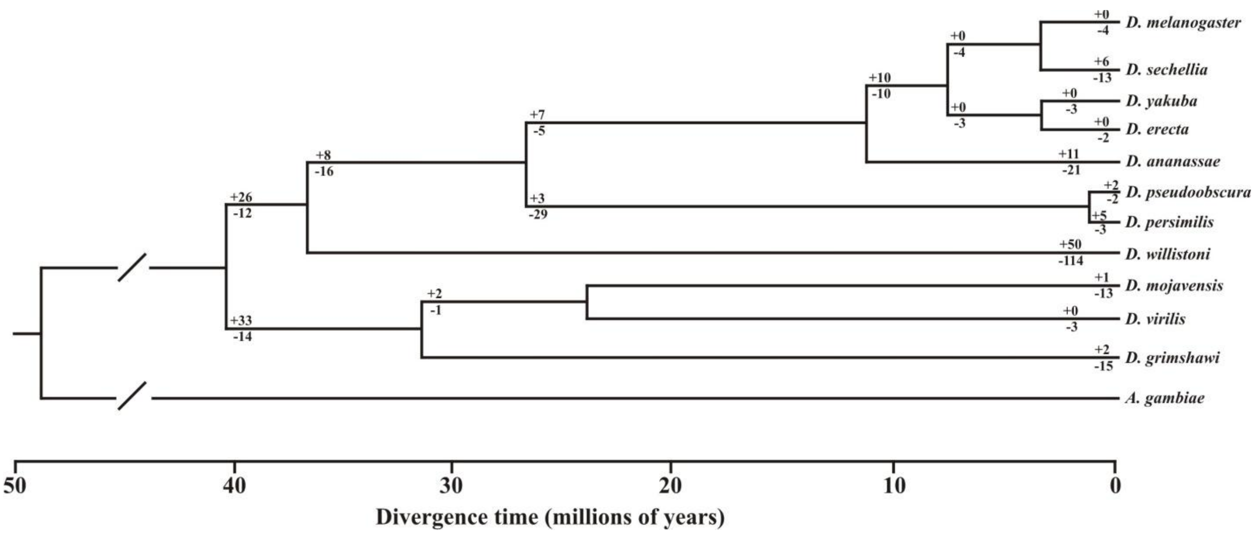

Figure S2. An example of intron sliding. (a) Global alignment of orthologous genes. All introns shown in this alignment are the fourth introns from their respective genes. Thirty shaded X’s represent introns. (b) Alignment and score between the intronic sequence of the fourth intron in Dwil\GK22863 and the fourth intron in Dper\GL17458.

(a)

```
Dmel\CG8026           ATCAAACAGACTTGGAG------------------------------GTACGAGCGCATG
Dsec\GM20618          ATCAAACAGACTTGGAG------------------------------GTACGAGCGCATG
Dyak\GE22456          ATCAAACAAACTTGGAG------------------------------GTACGAGCGCATG
Dere\GG10572          ATCAAACAGACTTGGAG------------------------------GTACGAGCGCATG
Dana\GF12007          ATCCGACAGACTTGGAG------------------------------GTACGAACGCATG
Dpse\GA20774          ATTAAACAGACTTGGAG------------------------------GTACGAACGCATG
Dper\GL17458          ATTAAACAGACTTGGAG------------------------------GTACGAACGCATG
Dwil\GK22863          ATCAAACAGACTTGGAGXXXXXXXXXXXXXXXXXXXXXXXXXXXXXXGTTTGAGGGCATG
Dgri\GH19865          ATCAAACAGACTTGGAG------------------------------GTACGAACGCATG
Dmoj\GI19815          ATCAAACAGACTTGGAG------------------------------GTACGAACGTATG
Dvir\GJ18531          ATCAAACAGACTTGGAG------------------------------GTACGAACGCATG

Dmel\CG8026           CGAGGTTTCTATAAGGGCCTG-GTGCCCTACCTGGTCCACGTCACGCCCAACATCTGCAT
Dsec\GM20618          CGAGGTTTCTATAAGGGCCTG-GTGCCCTACCTGGTCCACGTCACGCCCAACATCTGCAT
Dyak\GE22456          CGAGGTTTCTATAAGGGCCTG-GTGCCCTACCTGGTCCACGTCACGCCCAACATCTGCAT
Dere\GG10572          CGAGGTTTCTATAAGGGCCTG-GTGCCCTACCTGGTCCACGTCACGCCCAACATCTGCAT
Dana\GF12007          CGCGGATTCTATAAGGGCCTG-GTGCCCTACCTGGTTCACGTCACGCCCAATATCTGCAT
Dpse\GA20774          CGCGGTTTCTATAAGGGACTT-GTGCCGTACCTGGTCCACGTCACGCCCAACATCTGCAT
Dper\GL17458          CGCGGTTTCTATAAGGGACTT-GTGCCGTACCTGGTCCACGTCACGCCCAACATCTGCAT
Dwil\GK22863          CCCGGCTTCTATAAGGGATTGCAAGCCA-GTCTTGTGCGTGTGGT-CCCAGC--CTGTAT
Dgri\GH19865          CGTGGTTTCTATAAGGGACTC-GTGCCGTACTTGGTCCACGTAACGCCCAACATTTGCAT
Dmoj\GI19815          CGCGGTTTCTATAAGGGACTC-GTGCCGTACTTGGTCCACGTAACGCCCAACATTTGCAT
Dvir\GJ18531          CGCGGTTTCTATAAGGGACTA-GTGCCGTACCTGGTCCACGTAACGCCCAACATTTGCAT

Dmel\CG8026           G-----------------------------------------------------------
Dsec\GM20618          GXXXXXXXXXXXXXXXXXXXXXXXXXXXXXXCCTGCG---------TCTTTTCATTTG--
Dyak\GE22456          GXXXXXXXXXXXXXXXXXXXXXXXXXXXXXXCCTGCG---------TCTTTTCATTTG--
Dere\GG10572          GXXXXXXXXXXXXXXXXXXXXXXXXXXXXXXCCTGCG---------TCTTTTCATTTG--
Dana\GF12007          GXXXXXXXXXXXXXXXXXXXXXXXXXXXXXXCGCGTCCAACCAGCGTCTTTTCATTTG--
Dpse\GA20774          GXXXXXXXXXXXXXXXXXXXXXXXXXXXXXXCCAGAG---------TCTTTTAATTTG--
Dper\GL17458          GXXXXXXXXXXXXXXXXXXXXXXXXXXXXXXCCAGAG---------TCTTTTAATTTG--
Dwil\GK22863          G-----------------------------------------------------------
Dgri\GH19865          GXXXXXXXXXXXXXXXXXXXXXXXXXXXXXXACGAGC----------TTTTTCATTTCGC
Dmoj\GI19815          G-----------------------------------------------------------
Dvir\GJ18531          GXXXXXXXXXXXXXXXXXXXXXXXXXXXXXXGTAAGAG----------------------
```

(b)

```
E(10000): 1.5e-06; 57.6% identity (57.6% similar) in 774 nt overlap (144-849:10-695)

                  110       120       130       140       150       160       170       180
Dwil\GK22863: TAGAGGAAGTCCAAAATGCAGAATAGTATTAACCAGATGAATCTGCGGCAGAATTTAAGCACCTAGAATACATGAATCTA
                                                      ::::  :: ::::  : : :: :::: ::: :      ::
Dper\GL17458:                                GTCATGCTGATCT--GGGAGAAGCTGACCAGCTAG-ATAGA-----GTA

                                                     10          20        30              40

                  190       200       210       220       230       240             250
Dwil\GK22863: TCTATCAATCAATTTATTCTATATAGTTTTCGACCCTCTATTTTTTTTAAACATACAAAAAAGAA------AACATAGTA
              :  : ::   ::  ::   : :: : : :  ::      ::: :  :  :  ::    :: ::::       :: ::: :
Dper\GL17458: TTAACCA--GAACCTAGGATCTAGAATCTAGGA------ATTATGATCTAGAATCTGGAACAGAATTTAAGCACCTAGAA
                      50        60        70              80        90       100       110
```

```
                  260            270         280       290         300       310       320
Dwil\GK22863: TA----AGTCTATC-AG----CAAGAAAATG--ACAAAAGAAAACCAACAT--TATCTTATGAATTATTGTTACAAGTTC
              ::    : ::: :: ::    :   ::  ::   ::::    ::: :: ::   :: :: :: :::::::::: :: :::
Dper\GL17458: TACACGAATCTTTCTAGTTTCCTCCAATGTGCAGCAAACAGCAACAAAAATGACATGTTTTGTATTATTGTTATAA-TTC
                  120       130       140       150       160       170       180        190


                 330       340       350       360       370       380        390
Dwil\GK22863: GATGGATTTGACTAGTTTTGTTTTAATTATAATATTTAAATTAAGTGTATTCCATATATA-TGTGTAT------------
              :::  :    :: :  :             :::: :     :::::  : ::::::  :: : ::: :
Dper\GL17458: GATTAA--GAACCACCT------------AAATACTAGCCCTAAGTTAAATCCATAACTAGTTTGTTTTACGTTCTGCGC
                     200                   210       220       230       240       250


                     400       410       420       430       440       450       460       470
Dwil\GK22863: ATCTAAAC-TTGAAAGTGATTGTTGTGATAGCTGCACTAGCAATTGCAAAAGTTATGAAATCTAATTTCAAAGATGCATT
              ::::  :: :::::::: :::::::::::                      : ::::     ::: ::::::::::::::
Dper\GL17458: ATCTTCACGTTGAAAGTTATTGTTGTGAT----------------------GGTATG-----TAAATTCAAAGATGCATT
             260       270       280                             290            300       310

                         480       490        500       510       520       530       540
Dwil\GK22863: TCTT---CA-TTAACAAACGAAAACAACA-AAAACAAAAAACACGCACACAACATTTAGTTTATAGTTTAGTTAAATTAT
              ::::   :: :: ::   :: :: ::::: : : : : : :::: ::   :     :::::  ::::: : :: : ::::
Dper\GL17458: TCTTGCACACTTCAC---CG-AAGCAACACACAGCCACACACACACATTTAGTTCATAGTT--TAGTTAAATT-ATTTAT
                    320           330       340       350       360         370        380

                550         560        570                    580       590       600
Dwil\GK22863: TTATTTAAT--AATGAAATTGT-TACACA----AAACAA---------TAAGAAAAGAAAAAAATTTCATTTTGGCAAAA
              :: : ::::  : :: ::::::  :: ::    :::: :         : ::   :: : :::::::: :::::::::::
Dper\GL17458: TT-TGTAATGGATTGGAATTGTAAACGCAGCTGAAACCAGTTTCTAGTTTAGTTGAG-ACAAAATTTCGTTTTGGCAAAA
                  390       400       410       420       430       440        450       460


            610       620       630       640          650       660       670       680
Dwil\GK22863: AGATTCTTCATAATTTTGCATATGGTTTTATAATTTT---TATAAACTTGACATACAATATTTATGTAAATAAGCATAAA
              :::::::::::: ::: ::: :::: :::::::: ::    :: :: :  :: :   : : :::::::::::::::::::
Dper\GL17458: AGATTCTTCATAGTTTCGCAAATGG-TTTATAATCTTAAGGATGAAATGAACCT-TGACACTTATGTAAATAAGCATAAA
                  470       480        490       500       510        520       530       540

               690       700        710       720         730          740       750       760
Dwil\GK22863: AGTAAATCAAATCGCATTGTGAA-CAAATACGAAAAACC--AAGCAAAAAA---CAAATGAAACAAGTGACAGCAAATTG
              :           ::::::::::: ::   :: ::    :  ::::: :: :   : ::: :::: :   ::: : ::  :
Dper\GL17458: A-----------CGCATTGTGAACCACCAACTAAGCTGCTGAAGCAGAACAATCCCAATCAAACCA--TACAACCAAGAG
                                528       560       570       580       590         600

                    770       780       790        800         810        820       830
Dwil\GK22863: AATCCTAACAAAAAAGAAAAGAGAATTACTTA-ATTACAAT--TATGCTTAACTAT-TAAAATGCCAGGAAGGCAAAAAC
              ::    :: : : :: :  ::  : :::: :: :::: :::   :::  :  : ::  :::: : ::  ::   ::::::
Dper\GL17458: AA---GAAAATACAAAACTAGTTACTTACCTATATTAAAATGCCATG-ATGGCAATCCAAAAGGACACTAA--AAAAAAC
                610       620       630       640       650        660       670         680

                840        850       860       870       880       890       900       910
Dwil\GK22863: AAGAAA-GAAAGAAAAAACTATCTTAAAGTCTCCGCTTGCAAGACGAGTCTTTTCATTTACACCTTTACCAACAAAAAAC
              :: :::   :::::
Dper\GL17458: AACAAACTGAAGAACGTCTCGGGTCCAA
```

Figure S3. An example of a global alignment of orthologous genes using artificial introns. Example of a global alignment of one group of orthologous genes. Thirty shaded X's represent an intron.

```
Dmel\CG7911          ATGGAGTCCGAGTCGTTTTATGXXXXXXXXXXXXXXXXXXXXXXXXXXXXXXGTGTCACA
Dsec\GM12196         ATGGAGTCCGAGTCGTTTTATGXXXXXXXXXXXXXXXXXXXXXXXXXXXXXXGTGTCACA
Dyak\GE23428         ATGGAGTCCGAGTCGTTTTATGXXXXXXXXXXXXXXXXXXXXXXXXXXXXXXGTGTCACA
Dere\GG11978         ATGGAGTCCGAGTCGTTTTATGXXXXXXXXXXXXXXXXXXXXXXXXXXXXXXGTGTCACA
Dana\GF16212         ATGGAATCCGAATCGTTCTACGXXXXXXXXXXXXXXXXXXXXXXXXXXXXXXGTTTTACG
Dpse\GA20679         ATGGAGTCCGAATCTTTTTATGXXXXXXXXXXXXXXXXXXXXXXXXXXXXXXGTGTTACA
Dper\GL13489         ATGGAGTCCGAATCTTTTTATGXXXXXXXXXXXXXXXXXXXXXXXXXXXXXXGTGTTACA
Dwil\GK13375         ATGGAGACAGAATCTTTTTATGXXXXXXXXXXXXXXXXXXXXXXXXXXXXXXGTGTTACG
Dgri\GH22165         ATGGAGTCCGAATCGTTTTACGXXXXXXXXXXXXXXXXXXXXXXXXXXXXXXGTGTTACG
Dmoj\GI21966         ATGGAGTCCGAGTCTTTTTACGXXXXXXXXXXXXXXXXXXXXXXXXXXXXXXGTGTTACC
Dvir\GJ14325         ATGGAGTCCGAATCTTTTTATGXXXXXXXXXXXXXXXXXXXXXXXXXXXXXXGTGTTACG

Dmel\CG7911          CTCAGCGAGAAGGAGGCCATCGCACAGTTCGAGGTCCCAGATGTACCCGAGGAGTACATC
Dsec\GM12196         CTCAGCGAGAAGGAGGCCATCGCACAGTTCGAGGTCCCAGATGTACCCGAGGAGTACATC
Dyak\GE23428         CTCAGCGAGAAGGAGGCCATCGCACAGTTCGAAGTCCCGGATGTGCCCGAGGAGTATATC
Dere\GG11978         CTCAGCGAGAAGGAGGCCATCGCACAGTTCGAAGTCCCTGATGTACCCGAGGAGTATATC
Dana\GF16212         CTCAGCGAGAAAGAGCCCATTGCACAGTTCGACGTTCCTGATATTCCGGAGGAGTATGTG
Dpse\GA20679         CTCAGCGAGAAAGACCCAATCGCACAGTTCGATGTACCCGATATTCCAGAGGAGTACGTC
Dper\GL13489         CTCAGCGAGAAAGACCCAATCGCACAGTTCGATGTACCCGATATTCCAGAGGAGTACGTC
Dwil\GK13375         CTGAGCGAGAAAGAGCCAATTGCACAGTTCGATGTTCCCGAAATTCCAGAGGAGTATATA
Dgri\GH22165         TTGAGTGAAAAAGAGCCAATTGCACAGTTCGATGTGCCGGAAATTCCAGAGGAGTATATA
Dmoj\GI21966         CTCAGTGAAAAGGAGCCAATTGCACAATTCGATGTGCCGGAAATTCCAGAGGAGTATATC
Dvir\GJ14325         CTGAGCGAAAAAGAGCCAATTGCACAGTTTGATGTGCCGGAAATACCAGAGGAGTACATA

Dmel\CG7911          GTCCACTCGCACAAGCTCATCATCAAACAGATTTCCCTCGGCCCAGAAGCGAAGACCGGC
Dsec\GM12196         GTCCACTCGCACAAGCTCATCATCAAACAGATTTCCCTCGGCCCAGAAGCGAAAACCGGC
Dyak\GE23428         GTCCACTCGCACAAGCTCATCATCAAACAGATTTCCCTCGGCCCCGAGGCAAAGACCGGC
Dere\GG11978         GTCCACTCGCACAAGCTCATCATCAAACAGATTTCCCTCGGCCCAGAGGCAAAGACCGGC
Dana\GF16212         GTCCACTCACACAAGCTCATCATCAAACAGATTTCCCTCGGGCCCGAGGCAAAGTCCGGC
Dpse\GA20679         GTCCACTCACACAAATTGATAATAAAACAGATCTCCCTTGGATCCGAAGCGAAGACGGGG
Dper\GL13489         GTCCACTCACACAAATTGATAATAAAACAGATCTCCCTTGGATCCGAAGCGAAGACGGGG
Dwil\GK13375         GTGCATTCACACAAGTTAATAATTAAACAGATTTCGCTGGGACACGAGGCAAAAACAGGC
Dgri\GH22165         GTACACTCACACAAACTGATCATCAAACAGATCTCCTTGGGACCCGAGGCGAAAACGGGC
Dmoj\GI21966         GTACACTCACACAAGCTGATCATCAAACAGATATCCCTGGGACCTGAGGCGAAGTCCGGC
Dvir\GJ14325         GTACACTCGCACAAGCTGATCATTAAACAGATATCACTCGGACCCGAGGCAAAATCTGGT

Dmel\CG7911          GAATTCAACGTTGTACAGXXXXXXXXXXXXXXXXXXXXXXXXXXXXXXGCGGAGACGAAC
Dsec\GM12196         GAATTCAACGTTGTACAGXXXXXXXXXXXXXXXXXXXXXXXXXXXXXXGCGGAGACGAAC
Dyak\GE23428         GAATTCAACGTTGTACAGXXXXXXXXXXXXXXXXXXXXXXXXXXXXXXGCGGAGACGAAC
Dere\GG11978         GAATTCAACGTTGTACAGXXXXXXXXXXXXXXXXXXXXXXXXXXXXXXGCGGAGACGAAC
Dana\GF16212         GAATTCAACGTGGTGCAGXXXXXXXXXXXXXXXXXXXXXXXXXXXXXXGCCGAGACAAAC
Dpse\GA20679         GAATTTAATGTTGTACAGXXXXXXXXXXXXXXXXXXXXXXXXXXXXXXGCGGAGACAAAC
Dper\GL13489         GAATTTAATGTTGTACAGXXXXXXXXXXXXXXXXXXXXXXXXXXXXXXGCGGAGACAAAC
Dwil\GK13375         GAATTCAATGTTGTACAGXXXXXXXXXXXXXXXXXXXXXXXXXXXXXXGCTGAAACGAAC
Dgri\GH22165         GAATTCAAT---------XXXXXXXXXXXXXXXXXXXXXXXXXXXXXXGCGGAAACAAAC
Dmoj\GI21966         GAATTCAACGTCGTTCAGXXXXXXXXXXXXXXXXXXXXXXXXXXXXXXGCGGAAACGAAC
Dvir\GJ14325         GAATTCAATGTTGTACAGXXXXXXXXXXXXXXXXXXXXXXXXXXXXXXGCGGAAACCAAC

Dmel\CG7911          ATAAACGACGATGGCGAGAAGAAAACCTTGAAGATCCCCATTGCCGTGTTGAAGGTCGGC
Dsec\GM12196         ATAAACGACGATGGCGAGAAGAAAACCCTGAAGATCCCCATTGCCGTGCTGAAGGTCGGC
Dyak\GE23428         ATAAACGACGATGGCGAGAAGAAGACGGTGAAGATCCCCATTGCCGTGCTGAAGGTCGGC
Dere\GG11978         ATAAACGACGATGGCGAGAAGAAGACAGTGAAGATTCCCATTGCCGTACTGAAGGTCGGC
Dana\GF16212         ATAAACGACGATGG------GGAGACGGTGAAGATTCCCATTGCCGTCCTGAAAGTGGGC
Dpse\GA20679         GTGAACGATGATGGTGAGAAAAAGACTGTGAAGATTCCGATAGCAGTTCTGAAGGTCGGT
Dper\GL13489         GTGAACGATGATGGTGAGAAAAAGACTGTGAAGATTCCGATAGCAGTTCTGAAGGTCGGT
Dwil\GK13375         GCTATTAACGAGGGTGAGAAAAAGACAGTGAAGATTCCCATAGCCGTCCTGAAAGTAGGC
Dgri\GH22165         GTGCACGATGACGGCGAGAAAAAGACAGTTAAAATTCCCATTGCGGTCTTAAAGGTTGGC
Dmoj\GI21966         GTGAACGACGACGGCGAGAAGAAGACAGTCAAAATTCCCATTGCTGTTCTGAAGGTTGGA
Dvir\GJ14325         GTGCACGACGACGGCGAGAAGAAGACAGTCAAAATTCCCATTGCAGTCCTAAAGGTTGGC
```

```
Dmel\CG7911        GAGACCCGTAGCTTAAGACCAAATGTCGAGTTCCCCAATGGATCAGTGACCTTCAAACTG
Dsec\GM12196       GAGACCCGTAGCTTAAGACCAAATGTCGAGTTTCCCAACGGATCAGTGACCTTCAAACTG
Dyak\GE23428       GAGACCCGTAGCTTAAGGCCAAATGTCGAGTTCCCCAACGGATCAGTGACCTTCAAACTG
Dere\GG11978       GAGACCCGTAGCTTAAGACCAAACGTAGAGTTCCCCAACGGATCAGTGACCTTCAAACTG
Dana\GF16212       GAAACCCGTAGCTTAAGGCCAAATGTTGAATTCCCCAACGGATCCGTGACATTCAAGCTG
Dpse\GA20679       GAAACGCGCAGCCTGAGACCAAATGTGGAGTTCCCAAATGGATCAGTCACTTTCAAACTG
Dper\GL13489       GAAACGCGCAGCCTGAGACCAAATGTGGAGTTCCCAAATGGATCAGTCACTTTCAAACTG
Dwil\GK13375       GAAACTCGTTGCTTGAAGCCAAATGTTGAGTTCCCCAACGGATCGGTCACATTTAAACTA
Dgri\GH22165       GAAACGCGCAGTTTGAGGCCAAATGTTGAGTTCCCCAATGGCTCCGTCACATTCAAGCTC
Dmoj\GI21966       GAAACTCGCAGCTTGCGACCAAATGTTGAATTCCCCAATGGATCCGTCACATTTAAGCTT
Dvir\GJ14325       GAAACTCGCAGTTTGAGGCCAAATGTTGAATTCCCCAATGGCTCCGTCACATTCAAACTG

Dmel\CG7911        GTGCAGGGAAGTGGACCCGTACACGTATGCGGCAAGXXXXXXXXXXXXXXXXXXXXXXXX
Dsec\GM12196       GTGCAGGGAAGTGGGCCCGTACACGTCTGCGGCAAGXXXXXXXXXXXXXXXXXXXXXXXX
Dyak\GE23428       GTGCAGGGAACTGGACCCGTCTACGTCTGCGGCAAGXXXXXXXXXXXXXXXXXXXXXXXX
Dere\GG11978       GTGCAGGGAACTGGACCCGTCTACGTCTGCGGCAAGXXXXXXXXXXXXXXXXXXXXXXXX
Dana\GF16212       GTGCAGGGCACTGGACCCGTCTATGTCTGTGGCAAGXXXXXXXXXXXXXXXXXXXXXXXX
Dpse\GA20679       GTGCAGGGCACTGGGCCGGTCTATGTCTGCGGCAAGXXXXXXXXXXXXXXXXXXXXXXXX
Dper\GL13489       GTGCAGGGCACTGGGCCGGTCTATGTCTGCGGCAAGXXXXXXXXXXXXXXXXXXXXXXXX
Dwil\GK13375       ATCCAAGGCTCCGGTCCAGTTTATGTGTGCGGCAAG------------------------
Dgri\GH22165       GTCCAGGGCACGGGGCCGGTCTATGTTTGTGGAAAGXXXXXXXXXXXXXXXXXXXXXXXX
Dmoj\GI21966       GTCCAGGGCACGGGGCCGGTCTATGTGTGCGGCAAGXXXXXXXXXXXXXXXXXXXXXXXX
Dvir\GJ14325       GTCCAGGGCACGGGGCCGGTCTATGTCTGCGGCAAGXXXXXXXXXXXXXXXXXXXXXXXX

Dmel\CG7911        XXXXXXGTCGAGATGAACTTTGGCGAGTTTGACGACGGT------CAGATTTACGAGGAG
Dsec\GM12196       XXXXXXGTCGAGATGAACTTTGGCGAGTTTGACGACGGT------CAGATTTACGAGGAG
Dyak\GE23428       XXXXXXGTGGAGATGAACTTTGGCGAGTTTGACGACGGT------CAGATTTACGAGGAG
Dere\GG11978       XXXXXXGTGGAGATGAACTTTGGCGAGTTTGACGACGGT------CAGATTTACGAGGAG
Dana\GF16212       XXXXXXACGGAGATGAATTTCGGAGAGTTCAACGATGGT------GAAATTTACGAAGAG
Dpse\GA20679       XXXXXXGCCGAGATGAACTTTGGCGAGTTCGACGACGGG------CAGATGTATGAGGAG
Dper\GL13489       XXXXXXGCCGAGATGAACTTTGGCGAGTTCGACGACGGG------CAGATGTATGAGGAG
Dwil\GK13375       ------GCGGAAATGAATTTCGGCGAGTTCGATGATGGTGATGGTCAAATGTATGAGGAT
Dgri\GH22165       XXXXXXGCTGAAATGAACTTGGGTGAATATGACGATGGT------CAGATGTATGAGGAG
Dmoj\GI21966       XXXXXXGCGGAAATGAACTTGGGTGAATATGACGATGGT------CAAATGTACGAGGAG
Dvir\GJ14325       XXXXXXGCGGAGATGAACTTGGGTGAATACGACGACGGG------CAAATGTACGAGGAG

Dmel\CG7911        TATTCGGACGAGGAGGAGGATAGCGAACTGGAATTC-GACGA-AGAAGCAGCTCCTCAGA
Dsec\GM12196       TATTCGGACGAGGAGGAGGATAGCGAACTGGAATTC-GACGA-AGAAGCAGCTCCTCAGA
Dyak\GE23428       TATTCTGACGAGGAGGAGGATAGCGAACTGGAATTC-GACGA-AGAAGCAGCTCCTCAGA
Dere\GG11978       TATTCGGACGAGGAGGAGGATAGCGAACTGGAATTC-GACGA-AGATGCAGCTCCTCAGA
Dana\GF16212       TATTCTGATGAGGAGGATGACAGCGAATTGGAATTT-GACGA-GGATGCAGCTCCACAGA
Dpse\GA20679       TACTCCGACGACGAAGAGGACAGCGA-TGGGGACTTCGACGA-GGACGCTGTGCCGCAGA
Dper\GL13489       TACTCCGACGACGAAGAGGACAGCGA-TGGGGACTTCGACGA-GGACGCTGTGCCGCAGA
Dwil\GK13375       TATTCAGATGAAGAGGAGGAGGA------GGAGTAC-GACGA-GG------TGCCGCAGA
Dgri\GH22165       TATTCGGATGAGGACGATGACAGCGA-T--GAGCTC-GATGATGAGAGTT-CGCCACAGA
Dmoj\GI21966       TATTCGGATGATGAAGATGACAGCGAGCTGGAGGCT-GAGGACGATTGCT-CTCCACAAA
Dvir\GJ14325       TATTCGGACGACGAGGATGACAGTGAATTGGAGCTT-GATGACGATTGTT-CACCACAGA

Dmel\CG7911        CGAACGGCAAGAGTAA---TAAGAAGAAGTAG
Dsec\GM12196       CGAACGGAAAGAGTAA---TAAGAAGAAGTAG
Dyak\GE23428       CGAACGGCAAGAGTAA---TAAGAAGAAGTAG
Dere\GG11978       CGAACGGCAAGAGTAA---TAAGAAGAAGTAG
Dana\GF16212       CGAATGGAAAGAGCAA---TAAAAAGAAGTAA
Dpse\GA20679       CAAACGGCAAGAGCAAAATTACAAAGAAGTAA
Dper\GL13489       CAAACGGCAAGAGCAAAATTACAAAGAAGTAA
Dwil\GK13375       CGAACGGCAAGAGCAA---TAAAAAGAAGTAA
Dgri\GH22165       CAAATGGCAAGAGCAT---AAAAAAGAAGTAA
Dmoj\GI21966       CAAATGGAAAGAGCAA---TAAAAAGAAGTAA
Dvir\GJ14325       CAAATGGCAAGAGCAT---AAAAAAGAAGTAA
```

Figure S4. An example of an intron excluded during manual inspection.
This intron was excluded from further analyses due to low sequence identity surrounding the intron-exon junctions in the global alignment. This was done as a precautionary measure to exclude potential false-positives. Thirty shaded X's represent an intron. This is a fragment from the global alignment of these orthologous genes; only the region surrounding the excluded intron is shown.

```
Dmel\CG11660        CCTGGCACAAG---TTATAGCAACCGCCAACGAAGAGGA---TGAACCGGAGGACGA--A
Dsec\GM24726        CCTGGCCCAAG---TTATAGCAACCGCCAACGAAGAAGAGGATGAGCCGGAGGACGA--A
Dyak\GE20191        CCTGGCACAAG---TTATAGAAACCGCCAACGAAGAGGA---TGAACCGGAGGACGA--A
Dere\GG13901        CCTGGCACAAG---TTATAGAAACCACCAACG---------------CAGAGGATGA--A
Dana\GF24528        CCTCGTTCAAG---TCCTTGAAGCCAACAGCGATGGTGAGGATGCAGATGAGGACGAC-A
Dpse\GA11126        ----GC----------------ACTGCCAACAAGAAGGA----AAAGTAGAAGCTGGTGA
Dper\GL16346        ----GC----------------AATXXXXXXXXXXXXXXXXXXXXXXXXXXXXXX-----
Dwil\GK10469        GATGGCTTCAAGTGCTTTAGAGGCTGTCAACGGACATGA---------GGAGGATGG---
Dgri\GH16627        -TCTCTGCCAA---C-----AAACTACCGA----------TCCTGGCGAGGAATCTG---
Dmoj\GI11584        ACTTA-GCCAG---A-----AAGTTATAGAGAGTGCAAT-TTCCGGCGAAGAAACGG---
Dvir\GJ11263        -CTTGTGCCAG---C-----AAATTATTGATAACACCAA-TCCGGCCGAGGAAACTG---

Dmel\CG11660        GAGCCAGAG----GAATAC-GAT---GAAGACGATTACGAC-G-ATATCGGA-GATGACT
Dsec\GM24726        GAGCCAGAG----GAATAC-GAT---GAAGAGGACTACGAC-G-ATGTCGGA-GATGACT
Dyak\GE20191        GAGCCCGAG----GAATAC-GAT---GAAGAGGACTACGAC-G-ATGTCGGA-GATGACT
Dere\GG13901        GAGGTCGAG----GAATAC-GAT---GAAGAGGACTACGAC-G-ATGTCGGA-GATGACT
Dana\GF24528        ATGTCGAAG-----GCTAT-GACTACGAGGAGGATGACTATGA-GGAT--GGCGATGACT
Dpse\GA11126        GGATCTGGATTACGACTAT-GAT---GAAGAAGACGACGACTACGCGCTGGGCGATGAAT
Dper\GL16346        ---------------------AT---GAAGAAGACCACGACTACGCACTGGGCGATGAAT
Dwil\GK10469        AGATGTGGA------TTAT-GAT---GATGACGATGAAGACTACGATTTAAGCGATGGCT
Dgri\GH16627        --CCTCAGACT----ATGCAGATGATGATGAAGATGACGACTACGATCTGGGCGATAACT
Dmoj\GI11584        --ATTCAGACT----ATGCCGATGAAGATGAAGACGATGCCTTTGATGTTGGTGACGACT
Dvir\GJ11263        --ATTCGGACT----ATGCGGATGAGTATGAAGACGACGATTACGACCAGGGCGACGACT
```

Figure S5. An example of an intron gain that was identified in a single species. An intron gain occurred in Dana\GF17707. This is the third intron in this gene. Thirty shaded X's represent an intron. This is a fragment from the global alignment of these orthologous genes; only the region surrounding the gained intron is shown.

```
Dmel\Hdac3           ACGATACTACAGCGTTAATGTGCCGCTAAAGGAGGGCATTGATGACCAGAGCTATTTTCA
Dsec\GM10631         ACGATACTACAGCGTTAATGTGCCGCTAAAGGAGGGCATTGATGATCAGAGCTATTTCCA
Dyak\GE25277         ACGATACTACAGCGTGAATGTCCCGCTAAAGGAGGGCATTGATGACCAGAGCTATTTCCA
Dere\GG10995         ACGATACTACAGCGTGAATGTGCCGCTAAAGGAGGGCATTGATGACCAGAGCTATTTCCA
Dana\GF17707         CCGCTACTACAGCGTGAACGTCCCTCTTAAAGAAGGAATCGATGATCAGAGCTACTTCCA
Dpse\GA27397         TCGATACTACAGCGTGAATGTGCCACTCAAGGAGGGCATTGACGATCAGAGCTACTTCCA
Dper\GL22163         TCGATACTACAGCGTGAATGTGCCACTCAAGGAGGGCATTGACGATCAGAGCTACTTCCA
Dwil\GK11850         TCGCTATTATAGTGTGAATGTTCCGCTTAAAGAGGGAATCGATGATCAGAGCTACTTTCA
Dgri\GH18996         GCGATATTATAGCGTCAATGTGCCGCTTAAGGAGGGCATCGATGATCAGAGCTATTTTCA
Dmoj\GI24497         TCGTTATTACAGCGTTAATGTTCCTCTCAAAGAGGGAATTGATGATCACAGCTATTTTCA
Dvir\GJ24563         CCGGTATTATAGCGTTAATGTGCCTCTCAAGGAGGGTATTGACGACCAGAGCTACTTTCA

Dmel\Hdac3           G------------------------------GTGTTCAAACCCATAATTTCTGCCATTAT
Dsec\GM10631         G------------------------------GTGTTCAAACCCATCATTTCGGCCATTAT
Dyak\GE25277         G------------------------------GTGTTTAAACCAATCATATCGGCCATTAT
Dere\GG10995         G------------------------------GTGTTCAAACCAATCATATCGGCCATTAT
Dana\GF17707         GXXXXXXXXXXXXXXXXXXXXXXXXXXXXXXGTGTTTAAACCCATTATTTCGGCCATCAT
Dpse\GA27397         G------------------------------GTCTTCAAACCCATCATCTCGGCCATCAT
Dper\GL22163         G------------------------------GTCTTCAAACCCATCATCTCGGCCATCAT
Dwil\GK11850         G------------------------------GTTTTTAAGCCCATCATTTCGGCCATAAT
Dgri\GH18996         A------------------------------GTCTTCAAACCGATCGTTTCAGCAATTAT
Dmoj\GI24497         A------------------------------GTGTTCAAACCAATAATTTCAGCGATTAT
Dvir\GJ24563         A------------------------------GTGTTCAAGCCGATCATTTCTGCGATTAT
```

Figure S6. An example of an intron gain that occurred at a node. An intron gain that likely occurred in the ancestor of *D. pse* and *D. per*. This is the first intron in both Dpse\GA19454 and Dper\GL25213. Thirty shaded X's represent an intron. This is a fragment from the global alignment of these orthologous genes; only the region surrounding the gained intron is shown.

```
Dmel\dbo           GTCCAGACCCTCCTTCCCGCCGCCTGCCTGCTTCAACTGGTTGAGATTCAG---------
Dsec\GM25560       GTCCAGACCCTCCTACCCGCCGCCTGCCTGCTTCAACTGGTTGAGATTCAG---------
Dyak\GE22281       GTCCAGACCCTCCTTCCCGCCGCCTGCCTGCTTCAACTGGTTGAGATTCAG---------
Dere\GG15931       GTCCAGACCCTCCTTCCCGCCGCCTGCCTGCTTCAACTGGTTGAGATTCAG---------
Dana\GF24095       GTCCAGACTCTTCTGCCGGCCGCCTGCCTCCTCCAGTTGGTCGAGATCCAG---------
Dpse\GA19454       GTGCAGACTCTGCTGCCAGCCGCCTGCCTGCTTCAGCTGGTGGAGATCCAGXXXXXXXXX
Dper\GL25213       GTGCAGACTCTGCTGCCAGCCGCCTGCCTGCTTCAGCTGGTGGAGATCCAGXXXXXXXXX
Dwil\GK15757       GTTCAGACTCTGTTGCCAGCCGCCTGCCTGCTACAGCTTGTTGAGATTCAG---------
Dgri\GH17090       GTGCAAACACTGCTGCCAGCCGCCTGCCTGCTGCAGTTGGTCGAAATTCAG---------
Dmoj\GI11691       GTACAGACGCTGCTGCCGGCCGCCTGCCTGCTACAGCTCGTAGAGATTCAG---------
Dvir\GJ11367       GTCCAAACGCTGCTACCAGCCGCCTGCCTCCTGCAGCTCGTCGAAATACAG---------

Dmel\dbo           ---------------------GACATCTGCTGCGAGTTCCTCAAACGGCAATTGGATCCC
Dsec\GM25560       ---------------------GACATCTGCTGCGAGTTCCTCAAACGGCAGTTGGATCCC
Dyak\GE22281       ---------------------GACATCTGCTGCGAGTTCCTCAAACGGCAGTTGGATCCC
Dere\GG15931       ---------------------GACATCTGCTGCGAGTTCCTCAAACGGCAGTTGGATCCC
Dana\GF24095       ---------------------GACATATGCTGTGAGTTTCTCAAGCGGCAACTGGATCCC
Dpse\GA19454       XXXXXXXXXXXXXXXXXXXXXGACATTTGCTGCGAGTTCCTCAAGCGACAACTGGACCCG
Dper\GL25213       XXXXXXXXXXXXXXXXXXXXXGACATTTGCTGCGAGTTCCTCAAGCGCCAACTGGACCCG
Dwil\GK15757       ---------------------GACATTTGCTGTGAGTTCCTCAAACGTCAGCTGGATCCG
Dgri\GH17090       ---------------------GATATATGCTGTGAGTTCCTCAAGCGCCAGCTCGACCCG
Dmoj\GI11691       ---------------------GATATTTGCTGCGAGTTTCTCAAGCGCCAACTCGATCCG
Dvir\GJ11367       ---------------------GACATATGCTGTGAGTTTCTCAAGCGTCAACTCGATCCG
```

Figure S7. An example of an intron loss that was identified in a single species. An intron loss that occurred in Dwil\GK22366. Thirty shaded X's represent an intron. This is a fragment from the global alignment of these orthologous genes; only the region surrounding the lost intron is shown.

```
Dmel\CG4770      TCGGCAAGGCGAAATTGGGAAGXXXXXXXXXXXXXXXXXXXXXXXXXXXXXXCTTGAGCA
Dsec\GM26874     TCGGGAAGGCGAAATTGGGAAGXXXXXXXXXXXXXXXXXXXXXXXXXXXXXXCTTGAGCA
Dyak\GE25635     TCGGCAAGGCTAAATTAGGGAGXXXXXXXXXXXXXXXXXXXXXXXXXXXXXXCTTAAGCA
Dere\GG23603     TCGGCAGGGCCAAATTGGGAAGXXXXXXXXXXXXXXXXXXXXXXXXXXXXXXCTTGAGCA
Dana\GF17628     TCGGCAAAGCAAAGTTAGGAAGXXXXXXXXXXXXXXXXXXXXXXXXXXXXXXATTAAGCA
Dpse\GA18419     TCAACAAGGCAAAGTTGGGCAGXXXXXXXXXXXXXXXXXXXXXXXXXXXXXXTCTGAGCA
Dper\GL12080     TCAACAAGGCAAAGTTGGGCAGXXXXXXXXXXXXXXXXXXXXXXXXXXXXXXTCTGAGCA
Dwil\GK22366     TCCACAAGTCTCGATTGGGCAG------------------------------CATTAGCA
Dgri\GH19617     TGCACAAGGCGCAGCTGGGCAGXXXXXXXXXXXXXXXXXXXXXXXXXXXXXXCATCAGCA
Dmoj\GI22436     TGCACAAGGCGGAGCTGGGCAGXXXXXXXXXXXXXXXXXXXXXXXXXXXXXXCATCAGCG
Dvir\GJ10999     TGCACAAGGCAGAGCTGGGCAGXXXXXXXXXXXXXXXXXXXXXXXXXXXXXXCATCAGCA
```

Figure S8. An example of an intron loss that occurred at a node. An intron loss that likely occurred in the ancestor of *D.ere, D. yak, D. sec,* and *D.mel* but not *D. ana*. Thirty shaded X's represent an intron. This is a fragment of the global alignment of these orthologous genes; only the region surrounding the lost intron is shown.

```
Dmel\CstF-50          CTGATGATCAG------------------------------CCAGCTGATGTACGACGGC
Dsec\GM16423          CTGATGATCAG------------------------------CCAGCTGATGTACGATGGC
Dyak\GE23306          CTAATGATCAG------------------------------CCAGCTGATGTACGACGGG
Dere\GG11859          CTGATGATCAG------------------------------CCAGCTGATGTACGACGGG
Dana\GF23256          CTGATGATCAGXXXXXXXXXXXXXXXXXXXXXXXXXXXXXXCCAACTGATGTACGATGGC
Dpse\GA15331          CTGATGATAAGXXXXXXXXXXXXXXXXXXXXXXXXXXXXXXCCAGCTGATGTACGATGGC
Dper\GL23528          CTGATGATAAGXXXXXXXXXXXXXXXXXXXXXXXXXXXXXXCCAGCTGATGTACGATGGC
Dwil\GK10973          TTGATGATAAGXXXXXXXXXXXXXXXXXXXXXXXXXXXXXXTCAGTTGATGTACGATGGA
Dgri\GH18935          TTAATGATAAGXXXXXXXXXXXXXXXXXXXXXXXXXXXXXXCCAGTTGATGTACGATGGC
Dmoj\GI24451          TTAATGATAAGXXXXXXXXXXXXXXXXXXXXXXXXXXXXXXCCAATTAATGTACGATGGC
Dvir\GJ24120          TTAATGATAAGXXXXXXXXXXXXXXXXXXXXXXXXXXXXXXCCAGTTAATGTACGATGGC
```

Figure S9. Alignments that required the distant outlier *A. gambiae* to determine the ancestral state of the intron with Dollo parsimony. Thirty shaded X's represent an intron. These are fragments of the global alignment of these two groups of orthologous genes; only the region surrounding the intron in question is shown. (a) An example that required the ancestral state of the gene at this position, in this case intronless, to be known to infer that there was an intron gain in the ancestor of *D. gri*, *D. moj* and *D. vir*. (b) An example that required the ancestral state of the gene at this position, this case intron present, to be known to infer that there was an intron loss in the ancestor of *D. gri, D. moj* and *D. vir*.

(a)

```
Dmel\CG1332         TTCACCTTTGTGGACTTGAGCCCGCCAACAGAGGATGGTTCCAAATACGATTTCAG----
Dsec\GM14636        TTCACCTTTGTGGACTTGAGTCCGCCAACGGAGGATGGTTCCAAATACGATTTCAG----
Dyak\GE21425        TTCACGTTTGTGGACTTGAGTCCGCCAACGGAAGATGGTTCCAAATACGACTTCAG----
Dere\GG15207        TTCACCTTTGTGGACTTGAGTCCGCCAACGGAGGATGGTTCCAAATACGACTTCAG----
Dana\GF10565        TTCACCTTCGTGGACCTGAGTCCTCCGACGGAGGATGGTTCCAAGCACGATTTCCG----
Dpse\GA12201        TTCACCTTCGTGGACCTGAGCCCGCCCACTGAGGATGGGTCCAAGCACGACTTCAG----
Dper\GL12837        TTCACCTTCGTGGACCTGAGCCCGCCCACTGAGGATGGGTCCAAGCACGACTTCAG----
Dwil\GK12734        TTCATTTTTGTTGATCTAAGTCCACTCTCGGAGGACTCATCGAAGCATGATTTTCGT---
Dgri\GH15934        TTTACCTTTGTGGATCTGAGTCCAGCCTCCGAGGATGGCTCCAAGCATGATTTCAGXXXX
Dmoj\GI16583        TTCACGTTCGTGGACATGAGTCCTCCAATGGAAGATGGCTCCAAGCATGATTTTAGXXXX
Dvir\GJ12835        TTCACCTTCGTCGATCTGAGTCCGCCCCTGGAGGATGGCTCCAAGCACGATTTCAGXXXX
Agam\558841.2       CTCATGTTCACGGAAATAGCATCGCTGAAGGATGACGGCCAGCGGCACGACTTTAA----

Dmel\CG1332         --------------------------GTTCTCCACCAACAAGATACGTGAGGTTCATCTG
Dsec\GM14636        --------------------------GTTCTCCACCAACAAGATACGTGAGGTTCATCTG
Dyak\GE21425        --------------------------GTTCTCCACCAACAAGATACGTGAGGTGCATCTG
Dere\GG15207        --------------------------GTTCTCCACCAACAAGATACGTGAGGTTCATCTG
Dana\GF10565        --------------------------ATTCTCCACGAACAAGATACGGGAAGTGCATCTC
Dpse\GA12201        --------------------------GTTCTCCACCAACAAAATACGCGAGGTCCATCTG
Dper\GL12837        --------------------------GTTCTCCACCAACAAAATACGCGAGGTCCATCTG
Dwil\GK12734        ---------------------------TTTCACATCAATAAAATTCGAGAAGTTCATTTG
Dgri\GH15934        XXXXXXXXXXXXXXXXXXXXXXXXXXATTTTCAATCAACAAAATTCGGGAGGTTCATCTG
Dmoj\GI16583        XXXXXXXXXXXXXXXXXXXXXXXXXXGTTTTCCATTAATAAAATACGTGAAGTTCATCTG
Dvir\GJ12835        XXXXXXXXXXXXXXXXXXXXXXXXXXGTTTTCCATCAACAAAATCCGTGAGGTTCATCTG
Agam\558841.2       --------------------------GTTTCCGATCGGTCAACTGCGCGAGCTGCACCTG
```

```
----------------------------------------------------------------------------------------
```

(b)

```
Dmel\rtet           ACGGXXXXXXXXXXXXXXXXXXXXXXXXXXXXXXGTGTAATCATGACTTTGCTGCAGGGA
Dsec\GM15064        ACGGXXXXXXXXXXXXXXXXXXXXXXXXXXXXXXGTGTAATCATGACTTTGCTGCAGGGA
Dyak\GE25706        ACAGXXXXXXXXXXXXXXXXXXXXXXXXXXXXXXGTGTAATAATGACTTTGCTGCAGGGA
Dere\GG24354        ACAGXXXXXXXXXXXXXXXXXXXXXXXXXXXXXXGTGTAATAATGACGTTGCTGCAGGGA
Dana\GF16496        ACAGXXXXXXXXXXXXXXXXXXXXXXXXXXXXXXGTATTATCATGACTCTGCTACAGGGA
Dpse\GA19110        ACAGXXXXXXXXXXXXXXXXXXXXXXXXXXXXXXGTGTTATTATGACTTTGATTCAGGGA
Dper\GL21785        ACAGXXXXXXXXXXXXXXXXXXXXXXXXXXXXXXGTGTTATTATGACTTTGATTCAGGGA
Dwil\GK14183        ACAGXXXXXXXXXXXXXXXXXXXXXXXXXXXXXXGTATTATTATGACCATACTTCAAGGA
Dgri\GH18255        ACGG------------------------------GAGTTGTGATGACTTTGCTGCAGGGT
Dmoj\GI23654        ACAG------------------------------GGCTCGTTATGACTCTGCTACAGGGA
Dvir\GJ23498        ACGG------------------------------GCCTCGTCATGACCCTGCTGCAGGGT
Agam\321745.3       ACAGXXXXXXXXXXXXXXXXXXXXXXXXXXXXXXGTGTACTGATGGCGCTACTGCAAGGT
```

References

1. Zdobnov EM, et al.: **Comparative genome and proteome analysis of Anopheles gambiae and Drosophila melanogaster.** *Science* 2002, **298:**149-159.
