# Supplementary material for: Mechanisms of intron gain and loss in Drosophila
Source: BMC Evol Biol. 2011 Dec 19;11:364. doi: 10.1186/1471-2148-11-364 (PMC3296678; doi:10.1186/1471-2148-11-364)
Supplement: Additional file 2 — Ortholog dataset. A matrix of all orthologs used in final analyses. Each ortholog group is listed on one line. [file 1471-2148-11-364-S2.PDF]

Dmel	Dana	Dere	Dgri	Dmoj	Dper	Dpse	Dvir	Dwil	Dsec	Dyak
eIF2B-alpha	GF16217	GG11983	GH22202	GI21971	GL13494	GA20657	GJ14332	GK14086	GM12201	GE10409
Lsd-1-PC	GF16261	GG11228	GH23614	GI10367	GL23261	GA10284	GJ22586	GK22418	GM26530	GE10394
rtet	GF16496	GG24354	GH18255	GI23654	GL21785	GA19110	GJ23498	GK14183	GM15064	GE25706
CG1332	GF10565	GG15207	GH15934	GI16583	GL12837	GA12201	GJ12835	GK12734	GM14636	GE21425
CG6028	GF16951	GG12544	GH19847	GI22296	GL23723	GA19309-PB	GJ24088	GK13005	GM23673	GE24064
CG13601	GF17980	GG12393	GH17511	GI10378	GL23345	GA12394	GJ22840	GK22621	GM23530	GE23911
SMC2	GF11775	GG22392	GH20475	GI21017	GL10652	GA10161	GJ21940	GK23290	GM20175	GE12281
Hop	GF20636	GG24711	GH11548	GI17037	GL25924	GA15447	GJ24688	GK18373	GM16733	Hop
ERp60	GF11803	GG22623	GH20405	GI19593	GL10245	GA24137	GJ18390	GK22252	GM20403	GE13492
CG6330	GF18559	GG12162	GH19373	GI22212	GL27259	GA19516	GJ24332	GK11445	GM10155	GE10606
CG9297-PB	GF18617	GG19820	GH14273	GI23164	GL23092	GA21679	GJ14480	GK14198	GM24131	GE26298
Hrd3	GF18742	GG12409	GH11624	GI22400	GL24274	GA10167	GJ24649	GK22768	GM23543	GE23928
exba	GF20763	GG10932	GH17376	GI23920	GL22291	GA15521	GJ24012	GK22654	GM10614	GE24159
mys	GF21297	GG19682	GH24161	GI15296	GL26731	GA23031	GJ17000	GK25655	GM11222	GE15757
CG10795	GF12097	GG20778	GH21182	GI20469	GL11844	GA10564	GJ22319	GK20744	GM15723	GE13716
CG4364	GF21889	GG24014	GH25074	GI18209	GL19215	GA18135	GJ14807	GK25349	GM12330	GE10391
CG11134	GF22600	GG17808	GH11851	GI14333	GL19864	GA10783	GJ19387	GK25216	GM17635	GE17104
PH4alphaEFB	GF22904	GG11753	GH14110	GI24860	GL13463	GA15946	GJ24502	GK14136	GM12882	GE10881
Sra-1	GF22994	GG16915	GH19133	GI10855	GL23243	GA18534	GJ14463	GK11135	GM24222	GE24298
ea	GF23198	GG16913	GH14281	GI24877	GL23240	GA18526	GJ24520	GK11130	GM24220	GE24296
CG2126	GF23247	GG11850	GH14120	GI10648	GL24189	GA15258	GJ23006	GK14106	GM16414	GE23297
loj-PD	GF23515	GG15320	GH15831	GI12612	GL15550	GA10530	GJ12734	GK17263	GM14754	GE21539
Scox	GF23635	GG24334	GH10243	GI15364	GL26535	GA21389	GJ17274	GK24460	GM18054	GE18713
Prp31	GF23690	GG15883	GH14529	GI13100	GL24782	GA19924	GJ13847	GK20402	GM25514	GE22227
st	GF23914	GG13559	GH15896	GI16547	GL17865	GA18101	GJ12800	GK16717	GM25639	GE19858
CG5027	GF24161	GG15964	GH15857	GI12640	GL17836	GA18605	GJ12759	GK13693	GM25596	GE19532
Uch-L3	GF24661	GG14033	GH16209	GI13355	GL17965	GA17448	GJ13185	GK20334	GM24867	GE21236
Cypl	GF25039	GG14657	GH21768	GI22699	GL22598	GA12606	GJ23427	GK20536	GM14273	GE21017
CG7638	GF25243	GG15480	GH16926	GI13681	GL22761	GA20498	GJ14021	GK20335	GM25251	GE21790
CG3216-PC	GF12732	GG20817	GH21377	GI18553	GL10193	GA16724	GJ20348	GK23254	GM15763	GE13756
Aats-asp	GF12925	GG20342	GH21379	GI18555	GL17018	GA17710	GJ20350	GK20765	GM21429	Aats-asp
CG3608	GF12996	GG22994	GH20324	GI19996	GL10232	GA17555	GJ21245	GK21422	GM11887	GE14431
CG8386	GF13328	GG22310	GH19828	GI20416	GL10211	GA21037	GJ20088	GK10642	GM20100	GE14107

Cyp301a1	GF13394	GG20316	GH22036	GI19123	GL21304	GA21183	GJ22255	GK23301	GM21403	GE12475
CG8613	GF13496	GG20445	GH19740	GI20164	GL10685	GA21208	GJ22002	GK21469	GM21533	GE13577
CG8397	GF13574	GG22306	GH19824	GI20413	GL10208	GA21045	GJ20085	GK21889	GM20097	GE14104
CG15087	GF13761	GG20952	GH21850	GI20350	GL11316	GA13481	GJ22073	GK20712	GM19883	GE13891
CG4203	GF17837	GG16893	GH18976	GI22403	GL22140	GA18027	GJ10962	GK13302	GM24202	GE24275
porin	GF14059	GG10349	GH13331	GI17807	GL26379	GA19750	GJ17632	GK23973	GM11358	porin
CG8891	GF14164	GG24330	GH13806	GI18056	GL26487	GA21395	GJ22490	GK24597	GM18051	GE18710
CG31638	GF14167	GG10416	GH13651	GI13638	GL25673	GA25386	GJ18336	GK18725	GM18633	GE13912
CG8665	GF14331	GG21301	GH13317	GI12253	GL26664	GA21245	GJ17852	GK18741	GM23412	GE12917
CG8475	GF14405	GG23470	GH11504	GI17000	GL26472	GA21103	GJ24248	GK23741	GM13146	GE11152
l_2_01810	GF14412	GG23756	GH13099	GI14185	GL16129	GA18795	GJ17506	GK24355	GM26698	GE18563
Cpr	GF14516	GG10405	GH10739	GI11531	GL25521	GA11069	GJ12806	GK14755	GM18621	GE13802
Tsp	GF14952	GG23594	GH11027	GI17544	GL25970	GA10921	GJ15486	GK24219	GM13946	GE18413
oho23B	GF14986	GG24892	GH25136	GI19989	GL26288	GA15559	GJ13417	GK24281	GM18373	GE18186
CG11107	GF12707	GG23275	GH21159	GI18757	GL11162	GA10763	GJ21780	GK21317	GM20947	GE19122
Nhe1-PB	GF15048	GG24685	GH11719	GI16224	GL13878	GA11457	GJ16207	GK24457	GM16702	GE16483
CG15412-PB	GF15683	GG24444	GH10469	GI15875	GL19521	GA13710	GJ10361	GK14597	GM18150	GE14886
RfC3	GF15785	GG10129	GH13305	GI18168	GL19009	GA25212	GJ14606	GK15259	GM18361	GE18941
CG33253	GF15940	GG19210	GH12518	GI14870	GL12979	GA23068	GJ19534	GK25171	GM22946	GE17775
Fibp-PD	GF10845	GG13367	GH16910	GI13666	GL15677	GA23500	GJ14001	GK17216	GM16090	GE22461
CG5412	GF16680	GG23967	GH17157	GI10581	GL13564	GA18864	GJ22938	GK22454	GM23119	GE25670
CG3822	GF17009	GG15059	GH17276	GI22149	GL23254	GA17711	GJ24267	GK22690	GM23151	GE25031
CG14905	GF17068	GG16817	GH14685	GI13096	GL24529	GA13343	GJ13844	GK13857	GM15416	GE26136
CG6293	GF17530	GG17290	GH18455	GI24032	GL23836	GA19493	GJ23662	GK14417	GM26175	GE24691
Arc42	GF17623	Arc42	GH16400	GI22432	GL12205	Arc42	GJ10995	Arc42	GM26869	GE25631
CG8379	GF17691	GG13434	GH23943	GI10054	GL21552	GA21032	GJ23789	GK11970	GM23769	GE25913
Nmdar1	GF17704	GG10981	GH18993	GI24494	GL22160	GA15505	GJ24560	GK23704	GM10627	GE25274
CG1607	GF17783	GG11805	GH16486	GI22204	GL27248	GA27017	GJ24324	GK22762	GM12940	GE10940
CG3446	GF19540	GG17677	GH24919	GI15405	GL26776	GA17457	GJ16359	GK19996	GM12577	GE16467
CG32528	GF20217	GG19255	GH17695	GI16102	GL15018	GA22568	GJ15752	GK25282	GM22992	GE15874
Tsp5D	GF20316	GG17700	GH24112	GI16371	GL14958	GA22293	GJ16743	GK17445	GM12598	GE16487
rap	GF20850	GG18523	GH17898	GI15421	GL14550	GA15568	GJ16380	GK25147	GM12670	GE16840
Ahcy13	GF21238	GG17878	GH24475	GI15339	GL14580	GA11121	GJ16855	GK19859	GM19564	GE17184
CG2774	GF21580	GG24942	GH11581	GI17151	GL19242	GA15459	GJ17655	GK24153	GM11137	GE18233

Taf11	GF21885	GG24011	GH25071	GI18207	GL19212	GA17941	GJ14786	GK24821	GM12298	GE10358
CG3036	GF23558	GG25027	GH11190	GI18111	GL18642	GA15786	GJ16298	GK24471	GM18500	GE18317
Kap-alpha1	GF23594	GG16059	GH14657	GI11643	GL15735	GA21156	GJ11324	GK17499	GM19652	GE19625
gdl	GF23703	GG15903	GH14543	GI13114	GL24834	GA17270	GJ13862	GK15388	GM25533	GE22244
CG14830	GF23864	GG14985	GH15509	GI16786	GL21193	GA13281	GJ12528	GK16674	GM13779	GE20431
CdsA	GF23961	GG14472	GH16172	GI13325	GL22610	GA20725	GJ13151	GK20000	GM25022	GE21660
Sc2	GF24232	GG15168	GH15767	GI12546	GL16056	GA10597	GJ16064	GK16849	GM14597	GE21388
Faa	GF24408	GG14224	GH17004	GI11418	GL16789	GA13410	GJ13614	GK13735	GM14018	GE20652
path	GF24657	GG14028	GH15148	GI12085	GL17962	GA17443	GJ13356	GK10461	GM24863	GE21231
pyx	GF25054	GG14677	GH13730	GI23718	GL16103	GA14343	GJ20915	GK11308	GM14293	GE21038
CG1598	GF11712	GG10733	GH21552	GI19524	GL20106	GA14038	GJ21093	GK17805	GM20779	GE23838
TER94	GF11135	TER94	GH20288	GI19458	GL11509	GA15351	GJ21209	GK21952	GM21173	GE19324
Papst2	GF10102	GG13603	GH16468	GI12140	GL16196	GA20635	GJ13419	GK20462	GM25685	GE19898
CG10483	GF10434	GG14101	GH16888	GI12031	GL21369	GA10343	GJ13302	GK17527	GM13885	GE20523
CG5009	GF11673	GG21822	GH22025	GI19114	GL17222	GA18591	GJ22244	GK22892	GM21823	GE11899
Thiolase	GF12060	GG19986	GH20195	GI20722	GL10924	GA18273	GJ20459	GK15863	GM15500	GE11519
cdc2rk	GF12911	GG25234	GH19966	GI20253	GL10557	GA12412	GJ20206	GK21358	GM20555	GE21808
CG6459	GF13236	GG21031	GH21807	GI20778	GL17681	GA19611	GJ20513	GK15662	GM19961	GE13974
CG8314	GF13331	GG22313	GH19831	GI20419	GL20650	GA20978	GJ20091	GK22100	GM20103	GE14111
CG8306	GF13461	GG22258	GH22712	GI18498	GL11827	GA25137	GJ21361	GK10733	GM20048	GE14051
RpL23	GF13548	GG22807	GH21796	GI20545	GL10129	GA17595	GJ22396	GK20786	GM15964	RpL23
CG6153	GF14204	GG23793	GH11652	GI17954	GL19722	GA19395	GJ17725	GK18266	GM26892	GE18597
CG5142-PC	GF14217	GG23808	GH17741	GI22543	GL24735	GA18687	GJ10147	GK15311	GM10113	GE18614
CG10590	GF10453	GG14121	GH15015	GI13177	GL11985	GA10420	GJ11952	GK16877	GM13907	GE20545
CG7371	GF14751	GG24302	GH11452	GI21912	GL25904	GA20303	GJ17821	GK15054	GM18019	GE18997
CG10413	GF14838	GG21702	GH10529	GI17197	GL18742	GA10302	GJ19803	GK24654	GM17082	GE12725
CG15173	GF15216	GG21659	GH10833	GI17348	GL19222	GA13549	GJ16112	GK24841	GM17038	GE12679
CG10674	GF10475	GG14145	GH15278	GI11987	GL22472	GA10485	GJ12211	GK19814	GM13932	GE20574
cnir	GF15509	GG24462	GH10336	GI17095	GL19572	GA14423	GJ10862	GK14955	GM18171	GE14946
Mocs1	GF10488	GG15474	GH14930	GI11448	GL16733	GA17248	GJ13645	GK11297	GM25246	GE21783
Top2	GF15632	GG21630	GH10628	GI19824	GL21103	GA10169	GJ18099	GK24224	Top2	GE12650
CG5525	GF15661	GG10247	GH11666	GI17110	GL19739	GA18950	GJ16148	GK14617	GM26064	GE12130
CG9548	GF15704	GG23616	GH13247	GI17286	GL26100	GA21871	GJ17895	GK18690	GM17932	GE18435
CG5045	GF15771	GG10114	GH13604	GI20379	GL19149	GA18618	GJ13731	GK15263	GM18225	GE18928

CG31028-PC	GF16197	GG11957	GH18386	GI23378	GL14064	GA26944-PC	GJ10373	GK13146	GM12175	GE23406
CG9636-PC	GF16167	GG17469	GH19245	GI24280	GL23273	GA21931	GJ23059	GK14451	GM26363	GE24868
CG7911	GF16212	GG11978	GH22165	GI21966	GL13489	GA20679	GJ14325	GK13375	GM12196	GE23428
Plip-PB	GF16260	GG11227	GH23609	GI10365	GL23260	GA10281	GJ22584	GK22417	GM26529	GE10393
Osi2-PB	GF16375	GG13136	GH14018	GI24391	GL24055	GA11025	GJ14247	GK13033	GM10863	GE10186
CG5840	GF16410	GG22275	GH13554	GI24792	GL23580	GA19170	GJ24440	GK11036	GM15240	GE25476
CG12413	GF16439	GG12067	GH18719	GI22867	GL23093	GA29343	GJ14165	GK10998	GM16291	GE10510
CG16733	GF16584	GG15423	GH19934	GI22042	GL13941	GA14114	GJ24095	GK22430	GM23787	GE25933
chp	GF16594	GG11882	GH14339	GI10434	GL13952	GA14511	GJ10649	GK22554	GM12100	chp
pav	GF10567	pav	GH15541	GI16586	GL12840	GA11700	GJ12838	GK10551	GM14638	pav
CG6969	GF17004	GG11159	GH19219	GI22008	GL23247	GA19993	GJ10461	GK22572	GM26459	GE10324
CG5608	GF17038	GG17120	GH19003	GI10843	GL27170	GA19002	GJ14449	GK12108	GM26003	GE24512
CG34127-PB	GF17289	GG25051	GH19264	GI23471	GL12162	GA26209	GJ10226	GK11063	GM10464	GE25779
Aats-asn	GF15206	GG21647	GH13480	GI17232	GL21125	GA10495	GJ17991	GK24233	GM17026	GE12667
alpha-Est8	GF17300	GG25150	GH19271	GI23480	GL12173	GA10843	GJ10234	GK11080	GM10476	GE25786
TfIIFbeta	GF17437	GG17267	GH14638	GI23671	GL12621	GA19669	GJ23231	GK13842	GM26151	GE24669
pic	GF17579	GG19434	GH18378	GI23368	GL24391	GA20574	GJ10363	GK22405	GM24084	GE26244
CG7888-PB	GF10626	GG15470	GH14926	GI11443	GL15673	GA20662	GJ13640	GK11241	GM25242	GE21779
CG4770	GF17628	GG23603	GH19617	GI22436	GL12080	GA18419	GJ10999	GK22366	GM26874	GE25635
Hdac3	GF17707	GG10995	GH18996	GI24497	GL22163	GA27397	GJ24563	GK11850	GM10631	GE25277
KLHL18	GF17752	GG17168	GH12392	GI24060	GL21584	GA17529	GJ23685	GK11665	GM26048	GE24559
CG15706	GF12659	GG22290	GH21059	GI18661	GL16803	GA24300	GJ21677	GK20981	GM20079	GE14086
CG9363-PB	GF17762	GG17371	GH19138	GI23596	GL13668	GA21732	GJ23570	GK12126	GM26256	GE24775
CG3909	GF17851	GG17291	GH22558	GI23133	GL27199	GA17766	GJ10433	GK22499	GM23851	GE26002
Got1-PB	GF11770	GG20572	GH21676	GI18831	GL10236	GA24132	GJ21858	GK21961	GM21664	GE11758
CG17734	GF18164	GG17229	GH18805	GI10064	GL12612	GA14631	GJ23799	GK13290	GM26107	GE24630
CG14647-PB	GF18213	GG12384	GH14079	GI10612	GL21465	GA13145	GJ22966	GK13533	GM10753	GE25415
CG42388-PE	GF19188	GG18281	GH17799	GI15139	GL12974	GA23051	GJ19000	GK16231	GM13710	GE15814
CcapR-PC	GF18226	GG12210	GH18151	GI22912	GL22022	GA27348	GJ23325	GK14383	GM10207	GE10655
CG6073	GF18242	GG12196	GH18136	GI22898	GL22007	GA19338	GJ23311	GK13985	GM10193	GE10638
CG5205	GF18303	GG16932	GH19081	GI23225	GL23759	GA18736	GJ22884	GK11465	GM24241	GE24317
GluRIIE-PB	GF18326	GG15279	GH16113	GI22169	GL27141	GA16088	GJ24289	GK22710	GM23174	GE25052
CG14314	GF18512	GG22726	GH15763	GI23724	GL12598	GA12901	GJ23282	GK11249	GM15287	GE25520
Arpc3A-PC	GF18546	GG16957	GH19295	GI23512	GL22074	GA27369	GJ10263	GK12788	GM24264	GE24343

Rab23	GF18654	GG10890	GH18115	GI23830	GL23019	GA15247	GJ23289	GK14081	GM10600	Rab23
CG31120-PB	GF18678	GG12302	GH14258	GI23151	GL23076	GA27133	GJ10453	GK14273	GM16466	GE10757
Crc	GF18838	GG17350	GH19579	GI23963	GL21957	GA27323	GJ22515	GK13913	GM26236	GE24756
CG4596	GF18840	GG17803	GH18863	GI22933	GL12548	GA18286	GJ23217	GK13990	GM23910	GE26060
CG32687	GF19554	GG18917	GH12711	GI15063	GL14883	GA17074	GJ14718	GK17622	GM11308	GE15388
HspB8-PB	GF20408	GG19234	GH24938	GI11114	GL27055	GA12823	GJ15970	GK25198	GM22970	GE15853
CG8026-PB	GF12007	GG10572	GH19865	GI19815	GL17458	GA20774	GJ18531	GK22863	GM20618	GE22456
CG17896-PB	GF20808	GG12757	GH24555	GI16236	GL20166	GA14712	GJ15927	GK25041	GM19037	GE16583
Fer3HCH	GF20872	GG19524	GH24547	GI16260	GL16490	GA22605	GJ16902	GK25020	GM11516	GE16178
ade5	GF20891	GG19541	GH12574	GI14693	GL15026	GA17827	GJ16601	GK16398	GM11535	ade5
CG1402-PB	GF21031	GG19661	GH12585	GI16210	GL14732	GA26146	GJ15902	GK25008	GM11974	GE15736
Mcm3	GF21337	Mcm3	GH24613	GI11075	GL14679	GA18030	GJ14777	GK18502	GM12401	Mcm3
lic	GF21403	GG17753	GH24432	GI16291	GL16519	GA11504	GJ16530	GK25671	GM11599	GE17041
mRpL24	GF21543	GG24340	GH13595	GI23613	GL19491	GA21365	GJ20804	GK24587	GM18061	GE18719
sec5	GF21589	GG24944	GH11584	GI17153	GL19244	GA21362	GJ17657	GK23817	GM11139	GE18235
l_1_G0269	GF21841	GG17544	GH12888	GI14442	GL16570	GA14238	GJ18780	GK16309	GM22628	GE15304
CG9822	GF12100	GG20782	GH21186	GI20472	GL11847	GA22059	GJ22322	GK20747	GM15726	GE13719
CG10992	GF22391	GG19486	GH17748	GI15503	GL19846	GA10694	GJ19262	GK16352	GM17589	GE16138
Cdk5	GF22626	GG22344	GH20285	GI19456	GL11507	GA20894	GJ21207	GK21950	GM20132	GE14145
IP3K1	GF22812	GG10056	GH13823	GI20140	GL18935	GA17884	GJ13525	GK24866	GM17705	GE18870
CG7149	GF22846	GG23508	GH11383	GI18087	GL18851	GA20139	GJ16783	GK15304	GM13330	GE18335
Dbp45A	GF12227	GG23409	GH21617	GI20069	GL17616	GA11795	GJ21163	GK23245	GM21090	GE19250
CG2118	GF23246	GG11849	GH14119	GI10647	GL24188	GA15253	GJ23005	GK14105	GM16413	GE23296
CstF-50	GF23256	GG11859	GH18935	GI24451	GL23528	GA15331	GJ24120	GK10973	GM16423	GE23306
CG12069	GF23315	GG11917	GH14104	GI10636	GL13993	GA11372	GJ22992	GK13114	GM12138	GE23367
pds5	GF12284	GG20237	GH20852	GI20957	GL10519	GA14533	GJ20678	GK21335	GM21325	GE12396
CG6836	GF23655	GG16017	GH14607	GI13493	GL21723	GA19894	GJ11854	GK10391	GM15006	GE19582
CG9536	GF23668	GG23619	GH13250	GI17289	GL26104	GA21862	GJ17899	GK18693	GM17935	GE18438
CG9330	GF23707	GG16045	GH14627	GI13514	GL20943	GA21707	GJ11875	GK25825	GM19597	GE19611
Teh4-PB	GF23882	GG14214	GH15551	GI16826	GL12734	GA13421	GJ12574	GK10278	GM14006	GE20642
APP-BP1	GF23890	GG15477	GH15185	GI12125	GL25218	GA20612	GJ13397	GK12324	GM25249	GE21787
l_3_73Ah	GF23921	GG13566	GH15918	GI16570	GL17870	GA18020	GJ12822	GK20319	GM25646	GE19864
Sras	GF23981	GG15273	GH15194	GI12131	GL12258	GA18479	GJ13405	GK16882	GM14706	GE21494
CG13900	GF24063	GG14635	GH16029	GI12293	GL20829	GA12611	GJ13230	GK16677	GM14250	GE20993

dbo	GF24095	GG15931	GH17090	GI11691	GL25213	GA19454	GJ11367	GK15757	GM25560	GE22281
elgi	GF24099	GG15934	GH14771	GI11873	GL25216	GA14285	GJ13569	GK24430	GM25563	GE22867
Ide-PB	GF24144	GG13322	GH14596	GI13481	GL11914	GA18943	GJ11843	GK17230	GM22225	GE22411
Spt5	GF12341	GG21970	GH20202	GI20728	GL10931	GA20489	GJ20465	GK15869	GM21959	GE12049
CG11660	GF24528	GG13901	GH16627	GI11584	GL16346	GA11126	GJ11263	GK10469	GM24726	GE20191
CG34039-PB	GF24607	GG15685	GH14750	GI11491	GL21017	GA23804	GJ13685	GK10524	GM25469	GE22016
CG7509	GF24713	GG14161	GH15790	GI12570	GL17930	GA20402	GJ15474	GK17595	GM13949	GE20587
CG15019	GF24733	GG14188	GH15813	GI12595	GL17952	GA28349	GJ12716	GK11726	GM13977	GE20617
CG7970	GF24823	GG14812	GH15958	GI16611	GL11923	GA20730	GJ12865	GK24419	GM14435	GE21175
CG12026-PB	GF24845	GG14834	GH16164	GI13317	GL25316	GA11348	GJ12087	GK20164	GM14457	GE21197
Adk1-PB	GF24995	GG13846	GH15260	GI11969	GL25010	GA14347	GJ12193	GK13248	GM24672	GE20139
CG7979	GF25098	GG14475	GH15492	GI16768	GL22533	GA20738	GJ12512	GK11988	GM25024	GE21662
CycH	GF25171	GG16226	GH16374	GI11567	GL24864	GA20328	GJ11246	GK16840	GM22410	GE23026
CG12926	GF12489	GG25270	GH22856	GI21233	GL17330	GA11913	GJ20838	GK15622	GM20588	GE22148
CG11048	GF11750	GG22018	GH23034	GI21104	GL10167	GA10726	GJ20952	GK15906	GM21999	GE12095
mRpL2	GF25244	GG15481	GH16930	GI13684	GL22842	GA20496	GJ14025	GK20336	GM25252	GE21791
CG7741	GF12542	GG20188	GH21970	GI19673	GL17136	GA24978	GJ15039	GK22189	GM21276	GE12876
CG12164	GF12706	GG23272	GH21529	GI19500	GL11161	GA11448	GJ21071	GK21800	GM20945	GE19120
CG33958	GF12775	GG20983	GH20226	GI19397	GL16716	GA24277	GJ22459	GK23242	GM19918	GE13925
or	GF12884	GG22912	GH21700	GI20707	GL11643	GA15753	GJ19697	GK15725	GM16073	GE14351
CG5428-PB	GF13071	GG22874	GH20666	GI19029	GL16876	GA24321	GJ19995	GK23067	GM16034	GE14312
CG5222	GF10658	GG13497	GH14706	GI12252	GL25097	GA18747	GJ11484	GK23916	GM24446	GE19796
CG13188-PB	GF13107	GG20231	GH21480	GI19958	GL10094	GA25074	GJ17909	GK22050	GM21319	GE12392
swi2	GF13234	GG21029	GH20542	GI18811	GL17679	GA13020	GJ21838	GK21739	GM19959	GE13972
Rpn6	GF13517	GG22404	GH21149	GI18748	GL10628	GA24424	GJ21772	GK19517	GM20190	GE12293
Rs1	GF13667	GG23340	GH21077	GI18677	GL11206	GA15282	GJ21693	GK15774	GM21012	GE19181
CG17765	GF13767	GG20134	GH21258	GI19336	GL17295	GA14655	GJ22214	GK10715	GM21221	GE12824
CG7791	GF13838	GG10849	GH20228	GI19399	GL20060	GA20590	GJ22461	GK23013	GM16499	GE19442
TpnC41C	GF13927	GG10878	GH21731	GI18874	GL11049	TpnCIIIa	TpnCIIIa	GK21371	GM13554	GE11315
CG9987	GF14042	GG21180	GH13449	GI14026	GL21208	GA22169	GJ18257	GK23837	GM17346	GE13253
CG17218	GF14191	GG23775	GH11641	GI17943	GL18618	GA14397	GJ17714	GK15491	GM26756	GE18579
Ir25a-PB	GF14416	GG24350	GH13667	GI21042	GL18750	GA13857	GJ16133	GK24583	GM18071	GE14671
Taf13	GF14449	GG21587	GH13659	GI17411	GL26351	GA10547	GJ18344	GK18673	GM16962	GE12606
Cas	GF14542	GG20105	GH13775	GI17179	GL19330	GA12168	GJ22118	GK18154	GM17155	GE13161

vari-PB	GF14547	GG21549	GH10158	GI22442	GL19590	GA21703	GJ20335	GK24864	GM23306	GE13543
CG33123	GF14667	GG24431	GH13195	GI17774	GL19473	GA17300	GJ17599	GK14672	GM18143	GE14851
Tfb4	GF14734	GG24611	GH10566	GI21952	GL15043	GA18615	GJ17829	GK24367	GM16625	GE15755
CG10470	GF14694	GG21153	GH10893	GI13086	GL26158	GA10334	GJ11625	GK24167	GM17318	GE13226
Ppt2	GF14769	GG23717	GH13391	GI20683	GL19554	GA18478	GJ13278	GK21057	GM18996	GE18522
Max	GF10774	GG13417	GH14421	GI13548	GL20913	GA21938	GJ11907	GK19684	GM14884	GE22918
Fs_2_Ket	GF15351	GG21248	GH10879	GI18252	GL18516	GA15406	GJ23021	GK15231	GM23365	GE12343
RhoL	GF16059	GG17369	GH19398	GI22601	GL22229	GA21734	GJ23847	GK14302	GM26254	GE24773
rho-7	GF11795	GG22614	GH20399	GI19585	GL20022	GA21446	GJ18325	GK22246	GM20394	GE13484
twf	GF16237	GG16839	GH19702	GI23039	GL24238	GA16421	GJ24628	GK12190	GM24151	GE24220
CG13646	GF16317	GG11342	GH19450	GI22220	GL24405	GA12432	GJ24341	GK12978	GM26650	GE23537
tex	GF16672	GG17460	GH18366	GI10781	GL24122	GA21914	GJ14380	GK11705	GM26354	GE24859
Gfr	GF16675	GG17462	GH18913	GI23412	GL24125	GA21918	GJ10619	GK11707	GM26356	GE24861
Srp72	GF16682	GG23989	GH17177	GI10583	GL13566	GA18879	GJ22940	GK22456	GM23121	GE25672
CG7713	GF16853	GG22526	GH17286	GI23454	GL12394	GA20536	GJ23952	GK22662	GM15263	GE25499
Ranbp9	GF16860	GG18122	GH21659	GI22698	GL12000	GA18764	GJ23426	GK11292	GM23942	GE26093
CG5382-PB	GF16938	GG12524	GH19711	GI22279	GL24168	GA18838	GJ24073	GK14252	GM23659	GE24048
CG13827	GF16987	GG11196	GH18517	GI24362	GL24492	GA12553	GJ24372	GK11520	GM26496	GE10363
AP-1sigma	GF17027	GG11239	GH11883	GI24137	GL23383	GA19188	GJ24652	GK11486	GM26541	GE23431
Ir93a	GF17010	GG15081	GH17280	GI22152	GL23256	GA14432	GJ24269	GK22692	GM23153	GE25033
CG5641	GF17043	GG17126	GH17463	GI22787	GL27175	GA19024	GJ22789	GK12843	GM26008	GE24517
Cyp9f2	GF17048	GG17131	GH18868	GI24725	GL27180	GA26760	GJ14552	GK11195	GM26014	GE24523
Art1	GF17088	GG17264	GH19565	GI23860	GL21930	GA19682	GJ10573	GK13389	GM26148	GE24666
Ssl1	GF11005	GG16285	GH16776	GI13436	GL24973	GA10767	GJ11798	GK12882	GM22477	GE22646
Arp87C	GF17116	GG19181	GH21372	GI10502	GL24422	GA19410	GJ23183	GK13729	GM24057	GE26215
Art4	GF17144	GG17314	GH14177	GI22087	GL27288	GA18823	GJ24202	GK14504	GM26200	GE24716
atl	GF17217	GG11305	GH18437	GI24013	GL13613	GA19766	GJ23644	GK10964	GM26614	GE23502
RpII18	GF17248	GG12771	GH17834	GI22548	GL21641	GA11109	GJ11106	GK11847	GM10803	GE25464
pont	GF17460	GG17489	GH19121	GI23576	GL23687	GA17841	GJ23553	GK12828	GM23872	GE26023
HtrA2	GF17480	GG21285	GH13631	GI24802	GL24014	GA21097	GJ24448	GK12147	GM25850	GE26447
CG4860	GF17739	GG17152	GH15455	GI22465	GL21568	GA18485	GJ10063	GK11564	GM26034	GE24541
Ahcy89E-PC	GF17919	GG16804	GH18071	GI23750	GL23551	GA27300	GJ10596	GK13850	GM15398	GE26121
how	GF18066	GG11104	GH20998	GI10457	GL24013	GA10223	GJ23144	GK14241	GM26398	GE10266
CG2698	GF18152	GG14330	GH14051	GI24426	GL24454	GA15434	GJ14284	GK13068	GM10992	GE24959

CG10903	GF18155	GG14363	GH17335	GI10260	GL21481	GA10628	GJ11066	GK13473	GM10995	GE24963
cdm	GF18243	GG16707	GH17338	GI23878	GL12014	GA20183	GJ23968	GK11831	GM15325	GE25234
Elongin-B	GF18332	GG15357	GH16190	GI22177	GL13928	GA26968	GJ24297	GK22717	GM23182	GE25062
CG8032	GF18451	GG11620	GH17610	GI22532	GL23965	GA20779	GJ10135	GK13216	GM23747	GE25891
CG17565	GF18495	GG17001	GH19342	GI22551	GL21606	GA14558	GJ23741	GK13218	GM15154	GE24394
CG4546	GF18548	GG16959	GH23455	GI22950	GL22076	GA18246	GJ23766	GK11215	GM24266	GE24345
Aats-met	GF18774	GG21397	GH19679	GI24536	GL23293	GA16180	GJ22603	GK10894	GM25865	GE10041
CG34133-PB	GF18800	GG11704	GH16706	GI22303	GL14035	GA30040	GJ10490	GK13362	GM12834	GE23895
Karybeta3	GF18906	GG12549	GH19498	GI23769	GL21654	GA10419	GJ23951	GK11508	GM10770	GE25430
CG9914	GF19071	GG19334	GH12278	GI10976	GL21343	GA23040	GJ18534	GK25055	GM13412	GE11007
Marf-PB	GF19353	GG19591	GH24062	Marf	GL26855	GA17739	GJ17071	GK16527	GM12488	GE16751
CG6867	GF19364	GG19143	GH12447	GI14677	GL14936	GA19916	GJ16585	GK15168	GM22869	GE17702
CG5013	GF11913	GG16975	GH18761	GI22652	GL12217	GA18595	GJ23380	GK13257	GM24281	GE24363
CG3355	GF21354	GG25012	GH13131	GI22815	GL14423	GA17401	GJ23363	GK23752	GM18483	GE18301
Axs	GF21728	GG18235	GH11928	GI15349	GL12944	GA21976	GJ19453	GK19948	GM13377	GE15653
CG15449	GF21858	GG17559	GH12523	GI14877	GL16585	GA13737	GJ19540	GK10090	GM22643	GE15320
Fib	GF11304	Fib	GH21076	GI18675	GL10774	GA24481	GJ21690	GK19601	GM15587	Fib
CG4045	GF21935	GG12652	GH24451	GI21651	GL18335	GA17913	GJ16889	GK16167	GM18921	GE16980
CG4389	GF21951	GG24022	GH11058	GI17575	GL19219	GA18151	GJ17917	GK23812	GM12408	GE10479
CG9586-PB	GF22060	GG24037	GH13644	GI11923	GL25537	GA21893	GJ14035	GK15064	GM12539	GE10599
PGRP-SA	GF22064	GG18426	GH17605	GI16473	GL26887	GA11152	GJ15950	GK25449	GM13088	PGRP-SA
CG9281-PB	GF22241	GG19381	GH17634	GI16072	GL16523	GA21666	GJ15717	GK10328	GM22536	GE16028
Gas8-PB	GF22311	GG18578	GH24042	GI14903	GL15789	GA12872	GJ15335	GK16729	GM12721	GE16889
eIF3-S9-PB	GF11342	GG21791	GH20755	GI20840	GL11461	GA24735	GJ20575	GK21404	GM21793	GE11867
Tango13-PB	GF22576	GG17794	GH12501	GI14854	GL20242	GA26942	GJ19514	GK16105	GM17618	GE17090
mRpL22	GF22663	GG19077	GH24048	GI15151	GL26864	GA18397	GJ19341	GK14752	GM13465	GE17623
ppk28	GF21749	GG19083	GH11924	GI15345	GL16515	GA18445	GJ19317	GK25370	GM13471	GE17629
CG12241	GF11345	GG16895	GH18844	GI10106	GL12411	GA11502	GJ23843	GK11863	GM24204	GE24277
CG4935	GF22907	GG25196	GH11684	GI17131	GL16277	GA18537	GJ16167	GK24742	GM18660	GE21387
CG31019	GF22916	GG11764	GH14203	GI22112	GL13503	GA15943	GJ24229	GK14146	GM12895	GE10891
CG1635	GF22971	GG11815	GH18638	GI23398	GL23635	GA26673	GJ23089	GK11721	GM16432	GE10948
CG18347	GF23042	GG18542	GH23533	GI23551	GL22256	GA14898	GJ23511	GK11426	GM23990	GE26151
beta'Cop	GF23142	GG23847	GH13464	GI17217	GL26050	GA19792	GJ17974	GK14844	GM10466	GE18651
Rap2l	GF11365	GG22932	GH20176	GI20698	GL11660	GA24803	GJ20446	GK21923	GM18299	GE14369

mtSSB	GF23174	GG20113	GH18833	GI10091	GL12398	GA18119	GJ23828	GK14009	GM15451	GE26330
CG9471	GF23190	GG17327	GH14326	GI24882	GL23232	GA21813	GJ24524	GK11124	GM26213	GE24730
Rh2	GF23219	Rh2	GH14493	GI22968	GL23439	Rh2	Rh2	Rh2	GM17893	GE25563
CG6218	GF23238	GG20660	GH21802	GI21927	GL24295	GA19449	GJ14287	GK11957	GM25783	GE26388
CG5880	GF23277	GG11528	GH18968	GI23298	GL23902	GA27100	GJ24153	GK11281	GM10369	GE23718
CG1972	GF23370	GG11690	GH14325	GI10422	GL23717	GA15164	GJ10637	GK11920	GM12816	GE23879
Set	GF11410	GG16903	GH18852	GI22922	GL12537	GA18091	GJ23206	GK13791	GM24211	GE24285
CG13183	GF11799	GG22620	GH20403	GI19590	GL10242	GA12100	GJ18369	GK22250	GM20400	GE13489
CG6839	GF23654	GG16016	GH14605	GI13492	GL21712	GA19896	GJ11853	GK10387	GM15005	GE19581
Taf6	GF23708	GG16046	GH16937	GI11798	GL20945	GA16761	GJ13501	GK19071	GM19603	GE19612
Teh2	GF23881	GG14213	GH15550	GI16825	GL12733	GA13422	GJ12573	GK10267	GM14005	GE20641
CG6259	GF23900	GG15798	GH15350	GI12953	GL25031	GA19474	GJ13097	GK10800	GM24320	GE22134
CG7589	GF24205	GG13617	GH15188	GI12128	GL15583	GA23453	GJ13400	GK17743	GM25701	GE19913
fat-spondin	GF11435	GG20657	GH22839	GI21218	GL11519	fat-spondin	GJ20820	GK17866	GM21753	GE11642
CG9149	GF24451	GG14778	GH15449	GI13050	GL16175	GA21576	GJ12146	GK20386	GM14397	GE21141
CG32225	GF24473	GG13328	GH17191	GI13958	GL25041	GA16766	GJ13739	GK17236	GM22229	GE22417
CG6053-PB	GF24523	GG13896	GH14867	GI13720	GL16341	GA19324	GJ14064	GK10479	GM24721	GE20187
pex1	btl	GG15672	GH15109	GI13761	GL25293	GA23750	GJ13751	GK17038	GM25452	GE22001
Nmt	GF24703	GG14913	GH15985	GI16635	GL22566	GA20350	GJ12890	GK10839	GM24964	GE20367
Cdc37	GF24836	GG14824	GH16432	GI12516	GL25303	GA11342	Cdc37	GK20531	Cdc37	GE21187
Ak6	GF11470	GG22568	GH19842	GI20430	GL21288	GA21342	GJ20104	GK23083	GM20351	GE13438
Cpr65Ec	GF25006	GG14401	GH15387	GI12993	GL25022	GA23589	GJ13136	GK16649	GM14815	GE21591
TfIIEbeta	GF25060	GG15217	GH15942	GI16593	GL17873	GA11796	GJ12845	GK10558	GM14648	GE21436
Hexo1	GF25061	GG15218	GH15943	GI16594	GL17874	GA12099	GJ12846	GK10559	GM14649	GE21437
CG15020	GF25071	GG15228	GH15681	GI12727	GL17896	GA13439	GJ12714	GK16582	GM14659	GE21447
CG1707	GF12231	GG10756	GH20740	GI20823	GL17374	GA24807	GJ20556	GK22016	GM20801	GE24081
Tina-1	GF11524	GG19875	GH22048	GI19134	GL10895	GA15466	GJ22267	GK20729	GM11776	GE11399
CG9449-PF	GF10756	GG13404	GH14400	GI13526	GL20896	GA21794	GJ11885	GK19469	GM18426	GE22499
CG9350	GF11926	GG22078	GH20525	GI18971	GL10144	GA21720	GJ21995	GK23263	GM15797	GE12159
Bap55	GF11785	GG22198	GH20488	GI21032	GL10665	GA19676	GJ21958	GK17246	GM19984	GE14195
Rab2	GF11067	GG23219	GH21751	GI19298	GL11116	GA17076	GJ22175	GK23088	GM20894	GE19072
trsn	GF11105	GG22742	GH20630	GI20191	GL17199	GA11181	GJ20139	GK10677	GM20518	GE13101
CG4679	GF11198	GG22508	GH20279	GI19450	GL10414	GA24972	GJ21200	GK13197	GM20294	GE13378
Kdm4A	GF11231	GG10700	GH20585	GI19732	GL10386	GA13980	GJ18422	GK21350	GM20746	GE23530

CG2211	GF10062	GG14606	GH16228	GI13374	GL16071	GA28477	GJ13204	GK25450	GM14219	GE20966
Hsc70-5	GF11360	GG22433	GH20100	GI18996	GL10203	GA21150	GJ19961	GK20861	GM20220	GE12323
Gr64b	GF10086	GG15179	GH14696	GI13881	GL16986	Gr64b	GJ11735	GK17553	GM14611	GE21399
Cbp53E	GF11427	GG20649	GH22953	GI18443	GL11577	GA19795	GJ21527	GK19573	GM21744	GE11635
Buffy	GF11452	GG20214	GH21024	GI18624	GL10080	GA20921	GJ21633	GK17878	GM21300	GE12374
Ance-5	GF11532	GG19880	GH21978	GI19680	GL10903	GA10105	GJ17191	GK20737	GM11782	GE11404
CG4933	GF10115	GG15998	GH15886	GI16537	GL17856	GA18535	GJ12789	GK14080	GM25628	GE23088
TpnC73F	GF10103	GG13604	GH16402	GI12482	GL11919	TpnCIa	TpnCIa	GK15267	GM25686	GE19899
CG33056	GF10182	GG13255	GH16526	GI12206	GL24600	GA17253	GJ11439	GK17372	GM22160	GE22958
CSN3	GF10201	GG13277	GH15567	GI16842	GL26312	GA14892	GJ12591	GK13507	GM22181	GE22374
CG10863	GF10249	GG14235	GH15710	GI12765	GL16200	GA10606	GJ16106	GK16694	GM14027	GE20663
U3-55K	GF12083	GG22389	GH22986	GI18954	GL17526	GA10166	GJ21560	GK22122	GM20172	GE12278
Cpr66Cb	GF10288	GG14302	GH15054	GI12820	GL24694	GA20083	GJ12963	GK11921	GM25044	GE20730
RhoBTB	GF10335	GG16109	GH16972	GI11838	GL11881	GA19068	GJ13537	GK16960	GM22285	GE19673
CG5932	GF10345	GG16116	GH14550	GI13397	GL12789	GA19240	GJ11522	GK12291	GM22294	GE19683
CG10576	GF10449	GG14116	GH15010	GI13173	GL16317	GA10407	GJ11948	GK16873	GM13901	GE20541
CG11788	GF12257	GG22022	GH21819	GI20790	GL16838	GA11199	GJ20524	GK19536	GM22004	GE12100
Cyp6a22	GF12299	GG20487	GH21100	GI18701	GL10372	GA10183	GJ21720	GK21510	GM21577	GE13617
CG8258	GF12562	GG10613	GH22689	GI20616	GL10409	GA20937	GJ21056	GK21949	GM20657	GE22743
LvpH	GF12587	GG10641	GH20813	GI20901	GL10861	GA24513	GJ20632	GK21812	GM20686	GE23004
CG9416	GF12612	GG20912	GH21200	GI20486	GL10445	GA21772	GJ22338	GK20800	GM19836	GE13851
CG8090	GF12626	GG22371	GH20796	GI20881	GL11475	GA20816	GJ20616	GK19445	GM20156	GE12259
CG5482	GF12939	GG20961	GH21577	GI19549	GL11238	GA18915	GJ21121	GK21681	GM19892	GE13900
T3dh	GF13025	GG20700	GH20091	GI18989	GL16930	GA17444	GJ19950	GK20828	GM15646	GE11684
CG11208	GF13157	GG20879	GH22747	GI18529	GL10162	GA10842	GJ21395	GK15680	GM19803	GE13819
CG3215	GF13315	GG20063	GH23061	GI21131	GL11294	GA16715	GJ20981	GK19593	GM15578	GE11600
CG8315	GF13330	GG22312	GH19830	GI20418	GL20649	GA20979	GJ20090	GK22099	GM20102	GE14109
CG3776	GF13356	GG23023	GH19839	GI20427	GL11203	GA17681	GJ20100	GK19646	GM11918	GE14459
ClC-b	GF13396	GG20318	GH22038	GI19125	GL21306	GA21190	GJ22257	GK23152	GM21405	GE12477
CG12736	GF13674	GG10739	GH19814	GI20403	GL20115	GA11779	GJ20075	GK19666	GM20785	GE23915
Jheh1	GF13744	GG20936	GH19730	GI20154	GL10582	GA13492	GJ22431	GK20693	GM19863	GE13873
CG15100	GF13746	GG20938	GH19733	GI20156	GL10845	GA13491	GJ22433	GK20695	GM19865	GE13875
Aats-phe	GF13723	GG20415	GH22827	GI21206	GL20031	GA12223	GJ20807	GK19627	GM21501	GE12575
bwa	GF14446	GG21218	GH13657	GI17409	GL26347	GA12666	GJ18341	GK18730	GM17388	GE13293

CG8460	GF14623	GG10520	GH11509	moj30	GL18725	GA21094	GJ24303	GK15155	GM16819	GE18741
CG8455	GF14624	GG10521	GH11510	GI17006	GL18726	GA21091	GJ24314	GK15156	GM16830	GE18742
hgo	GF14763	GG23710	GH10766	GI20619	GL19546	GA18425	GJ13212	GK14896	GM18940	GE18517
Nle	GF14879	GG24639	GH10505	GI18023	GL19252	GA25887	GJ19693	GK24428	GM16656	GE16018
gammaTub23C	GF14993	GG24903	GH11085	GI17602	GL26300	GA16328	GJ17947	GK24850	GM18381	GE18195
CG10338	GF15067	GG21126	GH11606	GI17175	GL19630	GA10253	GJ17681	GK24688	GM17287	GE13200
CG2818	GF15249	GG24960	GH13702	GI23897	GL18669	GA15473	GJ21158	GK14645	GM18429	GE18251
Wwox	GF15334	GG23488	GH11466	GI16896	GL18508	GA20190	GJ17207	GK15005	GM13224	GE11246
CG2614	GF15348	GG21245	GH10876	GI18249	GL18513	GA15401	GJ22983	GK15051	GM23362	GE13319
SA	GF15436	GG10444	GH10376	GI24051	GL26195	GA28069	GJ21335	GK24253	GM16253	GE14220
CG12194	GF15492	GG25021	GH13224	GI18103	GL19373	GA11470	GJ16290	GK24465	GM18494	GE18310
Trs23	GF15539	GG25299	GH11277	GI17696	GL25559	GA21680	GJ11301	GK18753	GM17108	GE18790
yip2	GF15724	GG10063	GH11301	GI17721	GL18942	GA18290	GJ17549	GK18280	GM17755	yip2
CG6431-PB	GF15820	GG23668	GH10424	GI23465	GL26628	GA19587	GJ23963	GK14615	GM18713	GE18482
